# Effects of Age on Cortical Tracking of Word-Level Features of Continuous Competing Speech

**DOI:** 10.3389/fnins.2021.635126

**Published:** 2021-04-01

**Authors:** Juraj Mesik, Lucia Ray, Magdalena Wojtczak

**Affiliations:** Department of Psychology, University of Minnesota, Minneapolis, MN, United States

**Keywords:** speech perception, aging, electroencephalography, lexical surprisal, semantic processing, speech-in-noise (SIN) perception, temporal response function (TRF)

## Abstract

Speech-in-noise comprehension difficulties are common among the elderly population, yet traditional objective measures of speech perception are largely insensitive to this deficit, particularly in the absence of clinical hearing loss. In recent years, a growing body of research in young normal-hearing adults has demonstrated that high-level features related to speech semantics and lexical predictability elicit strong centro-parietal negativity in the EEG signal around 400 ms following the word onset. Here we investigate effects of age on cortical tracking of these word-level features within a two-talker speech mixture, and their relationship with self-reported difficulties with speech-in-noise understanding. While undergoing EEG recordings, younger and older adult participants listened to a continuous narrative story in the presence of a distractor story. We then utilized forward encoding models to estimate cortical tracking of four speech features: (1) word onsets, (2) “semantic” dissimilarity of each word relative to the preceding context, (3) lexical surprisal for each word, and (4) overall word audibility. Our results revealed robust tracking of all features for attended speech, with surprisal and word audibility showing significantly stronger contributions to neural activity than dissimilarity. Additionally, older adults exhibited significantly stronger tracking of word-level features than younger adults, especially over frontal electrode sites, potentially reflecting increased listening effort. Finally, neuro-behavioral analyses revealed trends of a negative relationship between subjective speech-in-noise perception difficulties and the model goodness-of-fit for attended speech, as well as a positive relationship between task performance and the goodness-of-fit, indicating behavioral relevance of these measures. Together, our results demonstrate the utility of modeling cortical responses to multi-talker speech using complex, word-level features and the potential for their use to study changes in speech processing due to aging and hearing loss.

## Introduction

Speech perception is fundamentally important for human communication. While speech signals are often embedded in complex sound mixtures that can interfere with speech perception via energetic and informational masking, the auditory system is remarkably adept at utilizing attentional mechanisms to suppress distractor information and enhance representations of the target speech (e.g., Ding and Simon, 2012a; Mesgarani and Chang, 2012; O’Sullivan et al., 2019). However, the robustness of speech perception, particularly in the presence of noise, is vulnerable to deterioration through both noise-induced and age-related hearing loss (Dubno et al., 1984; Helfer and Wilber, 1990; Fogerty et al., 2015, 2020) as well as age-related cognitive decline (van Rooij and Plomp, 1990; Akeroyd, 2008; Dryden et al., 2017). Additionally, a small but significant portion of the population experiences speech-in-noise (SIN) perception difficulties, without exhibiting clinical hearing loss (Saunders, 1989; Zhao and Stephens, 2007; Tremblay et al., 2015). Together, these SIN perception difficulties can lead to significant impairment in quality of life (Dalton et al., 2003; Chia et al., 2007), and in older adults they may result in increased social isolation (Chia et al., 2007; Mick et al., 2014; Pronk et al., 2014), potentially exacerbating loss of cognitive function (Loughrey et al., 2018; Ray et al., 2018).

Although subjective SIN perception difficulties are relatively common in older individuals, objective tests for quantifying these deficits, such as identification of words or sentences in noise (e.g., QuickSin; Killion et al., 2004), often do not strongly correlate with the degree of subjective deficit (Phatak et al., 2018), particularly in cases with little-to-no clinical hearing loss. Smith et al. (2019) recently reported that only 8% of their sample of 194 listeners exhibited deficits in objective SIN tasks, while 42% of listeners indicated experiencing subjective SIN perception difficulties. A likely reason for this mismatch is that objective speech perception tests do not accurately reflect real world scenarios where SIN difficulties arise. For example, while existing tests generally require identification of isolated words or sentences embedded in noise (e.g., speech-shaped noise or a competing talker), real world speech perception often requires real-time comprehension of multi-sentence expressions, embedded in a reverberant environment, in the presence of multiple competing speakers at different spatial positions. In these scenarios, listeners who need to expend additional time and cognitive resources to identify the meaning of the incoming speech may “fall behind” in comprehension of later parts of the utterance. Moreover, even if the listener can correctly piece together the meaning of the utterance, their subjective confidence may be diminished, potentially “blurring” the predictive processes thought to facilitate perception of upcoming speech (Pickering and Gambi, 2018). As such, behavioral measures that more accurately reflect subjective SIN perception difficulties may require utilization of more realistic, narrative stimuli, and focus on quantifying comprehension, as opposed to simple word or sentence identification (e.g., Xia et al., 2017).

While development of behavioral paradigms focusing on characterizing SIN perception difficulties is an important goal, a complementary and potentially more sensitive approach to quantifying these deficits may be provided by neural measures of continuous-speech tracking. In recent years, non-invasive methodologies for measurement of neural representations of continuous speech in humans have become increasingly popular (Lalor and Foxe, 2010; Crosse et al., 2016), particularly in application to young normal-hearing (YNH) populations. One important result of this work has been the demonstration of profound attentional modulation of speech whereby temporal dynamics of neural responses to attended and ignored speech differ considerably, both in representation of lower-level features such as the speech envelope (Ding and Simon, 2012a; Power et al., 2012; Kong et al., 2014; Fiedler et al., 2019), and higher-level features related to lexical and semantic content of speech (Brodbeck et al., 2018; Broderick et al., 2018). Indeed, while lower-level features produce robust responses even when speech is ignored, features related to linguistic representations only show robust responses for attended speech, suggesting that they are tightly linked with speech comprehension. Responses to higher-level features may therefore be particularly sensitive to SIN perception difficulties, which are likely associated with impaired comprehension performance. In fact, SIN perception difficulties could potentially manifest themselves not only in terms of poorer tracking of higher-level features in attended speech, but also in increased tracking of features in ignored speech, when facing difficulties with suppression of distractor information.

Changes in neural processing of continuous speech in aging populations, compared to young adults, are relatively poorly understood. Several studies have utilized magneto- and electroencephalography (M/EEG) to address this question. Studies comparing envelope-related cortical responses have revealed a pattern of amplified envelope representations in older populations (Presacco et al., 2016; Decruy et al., 2019; Zan et al., 2020), potentially reflecting changes in the utilization of cognitive resources during speech comprehension. More recently, Broderick et al. (2021) compared higher-level representations of speech in younger and older populations. They estimated EEG responses to 5-gram surprisal, reflecting the predictability of words given the preceding sequence of four words, as well as semantic dissimilarity, reflecting the contribution of each word to the semantic content of a sentence. While younger listeners showed strong responses to both of these features, older adults exhibited a delayed surprisal response and a near-absent response to semantic dissimilarity. These findings demonstrate that representations of higher-level features of speech may indeed reveal robust effects of age. However, because Broderick et al. (2021) did not report behavioral measures related to speech comprehension, nor measures of subjective speech perception difficulties among their participants, it is unclear whether these metrics would correlate with the reported EEG-based findings. Moreover, participants in that study were presented with clear speech without any distractors (e.g., competing speakers), making it unclear how speech representations differ in complex listening scenarios where speech perception difficulties are most commonly reported.

The goal of this study was to compare higher-level neural representations of two-talker speech mixtures between younger and older adults, and to explore how these measures relate to comprehension performance and self-reported SIN perception difficulties. In particular, we examined representations related to word dissimilarity relative to short-term preceding context, lexical surprisal based on multi-sentence context, and word-level audibility. We chose to pursue this paradigm for several reasons. First, a multi-talker paradigm was chosen because subjective SIN perception difficulties commonly arise in aging listeners in the context of competing speech. If age-related changes in neural representations are confirmed, then these neural signatures could potentially be further explored as a candidate objective correlate for subjective SIN difficulties. Second, we chose to characterize responses to word-level features linked to meaning and lexical predictability because existing evidence indicates that responses to higher-level features are tightly linked to speech comprehension (Broderick et al., 2018). As such, we anticipated that responses to these features are more likely to exhibit differences as a function of age and SIN perception difficulties. Although neural representations reflecting the end-goal of speech perception may allow for only limited inference about the underlying causes of SIN perception difficulties, which can range from peripheral changes in acoustic representations to more central changes in cognitive processes, these representations may offer increased sensitivity due to capturing the combined effects of the various etiologies underlying the deficit.

## Materials and Methods

### Participants

In total, 45 adult volunteers completed the experiment, and data from 41 participants were used due to a methodological change implemented early in data collection. The participant pool was divided into two groups, younger adults (YA) and older adults (OA), with participants who were 18–39 years included in the former, and participants who were 40–70 years included in the latter. The YA group consisted of 20 participants (6 male, 14 female; mean ± SD age: 29.40 ± 6.40 years), while the OA group included 21 participants (9 male, 12 female; mean ± SD age: 53.48 ± 8.68 years). Note that most participants in the OA group were not especially old (e.g., only five participants in the OA group were older than 60 years), and therefore the YA and OA group labels denote the relative age of these groups. Although our sample allows for treatment of age as a continuous variable, due to large individual differences in our measures, we decided to pursue binary classification of age (YA and OA) to increase the odds of detecting age-related differences between EEG response characteristics averaged across listeners in the two age groups. Participants were recruited via email advertisement from a pool of students, staff, and alumni of the University of Minnesota. All participants provided informed written consent and received either course credit or monetary compensation for their participation. The procedures were approved by the Institutional Review Board of the University of Minnesota.

### Audiometry

An air-conduction audiogram was measured in each ear for each participant prior to beginning the EEG procedures. Detection thresholds were measured at octave frequencies in the 250 – 8000 Hz range, and frequencies for which thresholds exceeded 20 dB HL were deemed to reflect hearing impairment (HI). This procedure resulted in the detection of 2 participants in the YA group, and 16 participants in the OA group as having mild-to-moderate high-frequency HI. The skewed distribution of HI toward the older population was expected, as hearing sensitivity naturally diminishes with age (see reviews by Huang and Tang, 2010; Yamasoba et al., 2013).

For participants with any hearing loss, all experimental audio materials were amplified in the frequency regions of hearing loss, as described in section “Stimuli” below. Under these conditions, we observed no association between task performance and high-frequency hearing loss.

### Modified SSQ Questionnaire

Prior to the EEG procedures, all participants completed a modified version of a subset of Speech, Spatial and Qualities of Hearing Scale (SSQ_m_). The original version of SSQ (Gatehouse and Noble, 2004) was designed to measure subjective hearing challenges faced by listeners in various situations of daily life. In our version, we specifically probed participants about difficulties with and frustrations related to hearing speech in noisy situations, such as cafes and social gatherings. Each of the 14 items was presented on a computer screen along with four graded choices of frequency, difficulty, or discomfort related to the presented listening scenarios. E.g.,

Item 1:

I find it difficult to talk with staff in places such as shops, cafes, or banks, due to struggling to hear what they are saying.

Item 10:

In group conversations I worry about mishearing people and responding based on incorrect information.

Response choices:

(1) Not at all
(2) Rarely
(3) Often
(4) Very often

### Stimuli

Stimuli were four public domain short story audiobooks (*Summer Snow Storm* by Adam Chase; *Mr. Tilly’s Seance* by Edward F. Benson; *A Pail of Air* by Fritz Leiber; *Home Is Where You Left It* by Adam Chase; source: LibriVox.org), spoken by two male speakers (two stories per speaker). Each story was about 25 min in duration and was pre-processed to truncate any silences between words that exceeded a 500-ms interval to 500 ms. On a block-by-block basis (see section “Experimental Procedures” below), each audiobook was root-mean-square (RMS) normalized and scaled to 65 dB SPL. Stimuli were presented to participants using ER1 Insert Earphones (Etymotic Research, Elk Grove Village, IL, United States), shielded with copper foil to prevent electrical artifacts in the EEG data.

In order to minimize the odds of finding age-related differences in neural responses that could be attributed to reduced audibility in participants with hearing loss, all audio materials were custom-filtered for each participant with HI. We used arbitrary magnitude FIR filters (order: 498) with linear-phase response, implemented in MATLAB (Mathworks, Natick, MA, United States; version R2019a) via the *designfilt* and *filter* functions. The filtering procedure introduced a constant group delay of ∼10 ms. The filter was designed to apply half gain, amplifying all frequency bands by half the amount of the hearing loss:

*A*(*f*) = 0.5×(*T*(*f*)−20) when T(*f)*> 20 dB HL,

*A*(*f*) = 0 otherwise,

where T(*f)* is the detection threshold in dB HL at frequency *f*. Note that half gain amplification is a commonly used strategy to mitigate reduced audibility due to hearing loss, while preventing discomfort from loudness recruitment, whereby loudness growth for frequencies affected by cochlear hearing loss is steeper than that observed in normal hearing (Fowler, 1936; Steinberg and Gardner, 1937).

### Experimental Procedures

**FIGURE 1:**
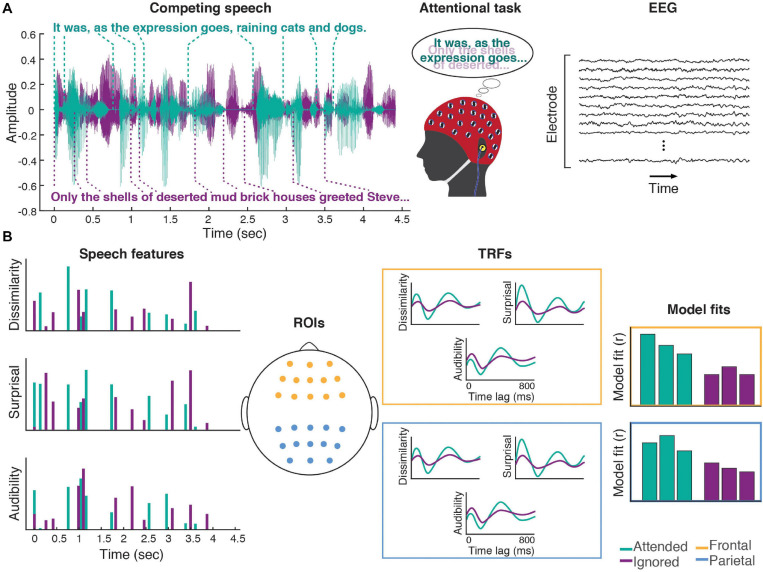
Experimental procedures. **(A)** Participants listened to a mixture of two speakers, while attending to one of them. Meanwhile, 64-channel EEG was recorded from their scalp. **(B)** Three word-level features (dissimilarity, surprisal, and audibility) were extracted from the speech for both the attended and ignored stories, and used to generate regressors containing impulses that were time-aligned to the word onsets scaled by the amplitude of each feature (note: word onset regressors and responses are not illustrated in the schematic). These features were regressed against the EEG signals recorded during the experiment, resulting in TRF and model fit contributions for each of the features. These TRFs and goodness-of-fit values were averaged across groups of frontal (yellow) and parietal (blue) electrodes for use in group-level analyses.

The experimental setup was implemented using the Psychophysics Toolbox (Brainard, 1997; Pelli, 1997; Kleiner et al., 2007) in MATLAB. Two experimental runs were completed by each study participant. In each run, a pair of audiobooks read by different male speakers (Figure 1A) was presented diotically (the mixture of the two audiobooks in each ear) to the participant. One of the stories served as the *attended* story, while the other was the *ignored* story, with these designations being counter-balanced across participants. A run was broken up into 24–27 blocks (variation was due to small differences in durations of audiobooks used in each of the two runs). Each block contained a roughly 1-min segment of audio, followed by a series of questions, detailed below. Block duration was allowed to exceed 1 min in order to ensure that each block concluded at the end of a sentence in the attended story. The attended story remained the same throughout the run. To cue the participants to follow the correct story, the audio of the attended story started 1 s prior to the onset of the ignored story. This was further aided by making this initial 1-s portion of the attended story in each block (except block #1) correspond to the final 1-s of the attended story from the previous block. These repeated segments with the attended story alone were excluded from statistical analyses. Throughout each block, participants were instructed to stay as still as possible, and to keep their gaze on a central fixation marker presented on a computer display in front of the participant. The purpose of this was to minimize EEG artifacts caused by muscle activity.

Following each block, participants were presented on a display with a series of Yes/No questions about the audio from that block, including:

(1) Four comprehension questions about the contents of the attended story
(2) Confidence ratings for each of the comprehension questions
(3) Intelligibility judgment about the attended speaker
(4) Subjective attentiveness rating

As each behavioral question had binary answer choices (e.g., for attentiveness, participants answered “Were you able to stay focused on the target story?” Yes/No), the main purpose of these questions was to gather information about participants’ comprehension and subjective experience throughout the run, and to make sure that they were attending to the correct story.

Participants were given 10 s to answer each question using a key press. If 10 s elapsed without a response, the question was marked as no-response. After answering each block’s questions, participants were allowed to request a short break to ensure that they remained comfortable throughout the experiment. These breaks were limited to up to 2 min, during which participants remained seated. The next block started as soon as the break was terminated by the participant with a key press, or 2 min elapsed. Furthermore, between the two experimental runs, participants were offered an extended break inside the booth. The EEG cap and the insert phones were not removed during the breaks.

The second experimental run was procedurally identical to the first one, except a different pair of stories was presented, neither of which was used in the first run. Additionally, the attended and ignored speakers were switched, so that the speaker that narrated the ignored story in the first run was attended in the second run, while the attended speaker from the first run became the ignored speaker in the second run. Participants were explicitly informed of this switch, and the purpose of this was to balance any possible speaker effects on each participant’s EEG data. The order of the story pairs in the two runs was counter-balanced across participants.

### EEG Procedures

While engaging in the experimental task described above, each participant’s EEG activity was sampled at 4096 Hz from their scalp using a Biosemi ActiveTwo system (BioSemi B.V., Amsterdam, Netherlands), with 64 channels positioned according to the international 10–20 system (Klem et al., 1999). Additional external electrodes were placed on the left and right mastoids, and above and below the right eye (vertical electro-oculogram, VEOG). Prior to the beginning of the recording, and between the two runs, the experimenter visually inspected signals in all electrodes, and for any electrodes with DC offsets exceeding ± 20 mV, the contact between the electrode and scalp was readjusted until the offset fell below ± 20 mV.

### EEG Preprocessing

All pre-processing analyses were implemented via the EEGLAB toolbox (Delorme and Makeig, 2004; version 14.1.2b) for MATLAB, unless otherwise stated. To reduce computational load, the raw EEG data were initially downsampled to 256 Hz, and band-pass filtered between 1 and 80 Hz using a zero-phase Hamming windowed sinc FIR filter (order: 846, transition band width: 1 Hz) implemented in the *pop_eegfiltnew* function of EEGLAB.

Subsequently, data were pre-processed using the PREP pipeline (Bigdely-Shamlo et al., 2015), in order to minimize the risk of signal contamination from noisy reference channels (e.g., due to poor electrode placement). Briefly, the pipeline includes three steps. First, line noise is removed using a multi-taper regression procedure implemented in the *cleanline* plugin (Mullen, 2012) for EEGLAB. Next, disproportionately noisy channels are detected via an iterative referencing procedure in which the data are initially referenced by an estimate of the global mean EEG activation, followed by detection of noisy channels via four data metrics. These include abnormal signal amplitudes, unusually low correlation with other channels, unusually poor predictability of channel data on the basis of other channels, and unusual degree of high-frequency noise. We utilized default parameters for each of the metrics, as outlined in Bigdely-Shamlo et al. (2015). After each iteration of this procedure, the noisy channels are excluded from being utilized in reference computation in the next iteration and the procedure is repeated up to four times or until channels identified as “noisy” don’t change across iterations. The noisy channels are finally replaced using EEGLAB’s spherical interpolation, and the final “clean” estimate of the global mean activation is used as the robust reference for the dataset.

Next, activations from all experimental blocks were epoched and independent component analysis (ICA; Jutten and Herault, 1991; Comon, 1994) was applied to the data using the infomax ICA algorithm (Bell and Sejnowski, 1995) implementation in EEGLAB. This procedure decomposes the EEG signal into statistically independent sources of activation, some of which reflect sensory and cognitive processes, while others capture muscle-related signal contributions and other sources of noise. We manually identified components that matched eye-blink related activity in component topography, amplitude, and temporal characteristics, as well as other high-amplitude artifacts that reflected muscle activity, and subtracted these components out of the data. This, on average, led to the removal of 2.52 (*SD*: 0.97) components.

The cleaned EEG signals were then band-pass filtered between 1 and 8 Hz with a Chebyshev type 2 filter designed using MATLAB’s *designfilt* function (optimized to achieve 80 dB attenuation below 0.5 Hz and above 9 Hz, with pass-band ripple of 1 dB), and applied to the data using the *filtfilt* function. This filtering was chosen due to prior evidence indicating that neural responses to continuous speech track predominantly the low-frequency fluctuations within the ∼1–7 Hz range (e.g., Ding and Simon, 2012b; Zion Golumbic et al., 2013). Afterwards, the data were *z*-scored in order to control for inter-subject variability in the overall signal amplitude due to nuisance factors such as skull thickness or scalp conductivity, as well as to improve efficiency in the cross-validated regression and ridge parameter search for deriving the temporal response function (TRF), described below (see section “TRF Analyses”). Finally, because run duration varied slightly due to unequal lengths of the two pairs of audiobooks (i.e., 24–27 min), in order to equalize contributions from each run to the overall analysis results, only blocks 2–23 from each run were used in the remaining analyses. The first block was excluded in order to minimize effects of initial errors in attending to the target story, which happened to a very small number of participants (less than 5), but was quickly corrected after initial comprehension questions were presented.

### Word Timing Estimation

Word onset timings for all words within each story were estimated using the Montreal Forced Aligner (McAuliffe et al., 2017). Prior to running the aligner, the audiobook text was preprocessed to remove punctuation, typographic errors and abbreviations, and both the text and audio were divided into roughly 30-s segments. This segmented alignment approach was used in order to prevent accumulation of alignment errors for later portions of the audio. All alignments were subsequently manually inspected for timing errors, and when noticeable alignment errors were detected, the aligner was re-run on further-shortened (15-s) segments of the affected audio. While forced alignment routinely results in some degree of timing errors, these are typically small, with a median of about 15 ms for the aligner used here. As such, only a small degree of temporal smearing of estimated neural responses should occur due to these errors.

### Data Analysis

#### TRF Analyses

Time courses of cortical responses to different speech features, known as the TRFs, were extracted from preprocessed EEG activity using cross-validated regularized linear regression, implemented via the mTRF toolbox (Crosse et al., 2016). Briefly, deconvolution of a TRF for a given feature from the EEG signal is accomplished by first constructing a regressor containing a time series, sampled at a rate matching the EEG signal, of that feature’s amplitudes. By including multiple time-lagged copies of the regressor for each feature, the effect of a given feature on the neural activity at different latencies relative to the word onset can be estimated, resulting in a time course of neural response. Regressors for all features are combined into a full design matrix, and this matrix is then regressed against the EEG signal to yield the impulse responses (i.e., TRFs) for each of the included features at each electrode site.

In practice, this procedure was implemented through 11-fold cross-validation, with each fold involving three steps. First, the data and regressors were split into a training set, composed of 40 blocks of the data (∼40 min), and a testing set, containing the remaining 4 blocks of the data (∼4 min). Next, the training set was used to determine the ridge parameter, λ, using leave-one-out cross-validation. On each iteration, 39 trials from the training set were used to fit the cortical-response model using a range of ridge parameters, and the resulting TRFs were then evaluated on their ability to predict the data in the remaining trial by computing the Pearson’s correlation coefficient between the predicted and actual EEG signal. The goodness-of-fit values for each λ were then averaged across all cross-validation folds and all electrodes, and the parameter with the best average fit was selected to be used for estimating the TRFs using all 40 training trials. These TRF estimates were then used to assess the model goodness-of-fit using the test data. This was done by convolving the estimated TRFs with the corresponding word-feature regressors for the test data set, and computing the Pearson’s correlation between the predicted and actual test data. Following cross-validation, average TRFs for each feature and an average model goodness-of-fit were computed from results of all cross-validation folds for use in group-level analyses.

##### Regression features

Word features used in the regression analyses included word onsets, semantic dissimilarity, surprisal, and word audibility (Figure 1B). All regressors included feature values for all words (both content and function words) in the speech stimuli. Word onset regressors contained unit-amplitude features aligned with word onsets, and their purpose was to account for lower-level activity elicited by the acoustic onset.

###### Semantic dissimilarity

Semantic dissimilarity, reflecting approximately the degree to which each word adds new information to a sentence, was computed as described in Broderick et al. (2018). Briefly, we used Google’s pre-trained *word2vec* neural network (Mikolov et al., 2013a, b), implemented using the Gensim library (Rehurek and Sojka, 2010) for Python, to compute a 300-dimensional vector representation (otherwise known as an embedding) of each word within our stimuli. An important property of these vector representations is that in the 300-dimensional vector space, vectors of words with similar meanings point in similar directions. Computing the correlation between vectors representing any two words approximates their semantic similarity. Because EEG response to incongruent words has been shown to elicit a strong N400 component (Kutas and Hillyard, 1980), for regression purposes these similarity values were subtracted from 1 to convert them to dissimilarity.

To construct semantic dissimilarity regressors, we computed the dissimilarity between each word’s vector, and the average of vectors for all preceding words in a given sentence. In the case of the first word in a sentence, we computed dissimilarity from the average vector for words in the previous sentence. These dissimilarity values were then used to construct the regressor consisting of unit-length impulses aligned to word onsets that were scaled by each word’s dissimilarity value and zeros between these impulses. Although neural responses to semantic content of words may not be strictly time-locked to word onsets, potentially leading to some degree of temporal smearing in the estimated TRFs, word onset timings have been successfully used as timestamps for characterizing higher-order lexical and semantic processes (e.g., Broderick et al., 2018; Weissbart et al., 2019).

###### Lexical surprisal

Surprisal regressors were constructed in an identical way to dissimilarity, except the feature values were computed using OpenAI’s GPT-2 (Radford et al., 2019; 12-layer, 117M parameter version) artificial neural network (ANN), similar to the approach demonstrated by Heilbron et al. (2019). These procedures were implemented in Python using the Transformers library (Wolf et al., 2020) for PyTorch (Paszke et al., 2019). GPT-2 is a transformer-based (Vaswani et al., 2017). ANN that, using a “self-attention” mechanism, is capable of effectively using hundreds of words worth of preceding context in order to generate seemingly realistic sequences of text. As a result, it can be used as a proxy for computing the predictability of words within a sequence. Surprisal is calculated based on a much longer time scale (a large number of words in the preceding context) than semantic dissimilarity. Specifically, by providing GPT-2 with a segment of text and then generating the distribution over the next word, it is possible to assess the relative probability of the actual next word within GPT-2’s distribution of possibilities. Generation of all probabilities involves iteratively adding words into the context, and computing the probability of each successive word. In practice, GPT-2 utilizes a tokenized representation of text, whereby GPT-2’s vocabulary corresponds to a combination of whole words (particularly in the case of shorter words) and word fragments.

As a result, the probability of the i-th word *w*_*i*_ was computed as a product of conditional probabilities of the constituent word tokens *t*, with each token’s probability being computed with the model’s knowledge of the preceding tokens (i.e., preceding text plus current word’s tokens whose probabilities were already estimated):

$$p\,\left(w_{i}\right)=\prod_{j=1}^{n}p\,\left(t_{k+j}|t_{k+j-512},\ldots \,t_{k+j-1}\right)$$

where *j* indexes the *n* tokens of word *w_*i*_, k* is the absolute index of the last token in the preceding word (relative to text beginning), and 512 is the maximum number of tokens utilized for prediction. For token indices less than 512 (i.e., early portions of the text), all of the available context was used. Furthermore, in cases where one or more tokens from the word at the far boundary of the context window did not fit into the 512 token limit, that word’s tokens were excluded from being used for prediction. Although GPT-2 is capable of utilizing up to 1024 tokens for prediction, we utilized a context length of 512 tokens due to limited computational resources. Across the four stories, when full predictive context was utilized for prediction, it contained on average 393.3 [*SD* = 31.1] words.

Because brain mechanisms underlying lexical prediction respond more to unexpected than to expected words (Kutas and Hillyard, 1984), surprisal was computed by taking the negative log of the conditional probabilities of each word, leading to less expected words receiving higher surprisal values:

$$S\,\left(w_{i}\right)=-\log_{10}\left(p\,\left(w_{i}\right)\right)$$

###### Audibility

Word audibility regressors were constructed separately for the attended and ignored stories to capture the degree of masking of each word in one story by the speaker of the other story. In contrast to dissimilarity and surprisal, this value reflects the information at the shortest, word-by-word time scale, with higher signal-to-noise ratio (SNR) values reflecting greater peripheral fidelity of target speech, leading to lower uncertainty in speech identification on the basis of the bottom-up signal. For each word *w*_*i*_ in a given story, its audibility was defined in dB SNR units:

$$A\,u\,d\,\left(w_{i}\right)=20\,\log_{10}\frac{R\,M\,S\,\left(y\,\left(w_{i}\right)\right)}{R\,M\,S\,\left(z\,\left(w_{i}\right)\right)}$$

where y(*w*_*i*_) is the acoustic waveform of a word *w*_*i*_ spoken by one speaker, and z(*w*_*i*_) is the acoustic waveform of the other speaker at the same time. Because neural responses have limited dynamic range while the audibility measure ranged from –inf to inf, the audibility values were rescaled to range from 0 to 1. In order to do this, audibility values were first clipped above 10 dB and below –10 dB, and then scaled to the 0–1 range by:

$$A\,u\,d_{s\,c\,a\,l\,e\,d}=\frac{A\,u\,d+10}{20}$$

Finally, because the distributions of regressor values had distinct means for different features, we scaled each feature’s non-zero regressor values to have an RMS of 1. Bringing different features into similar amplitude ranges was done in order to make the amplitudes of corresponding TRFs more similar to each other, thus improving regularization performance.

It is notable that although neither dissimilarity, nor surprisal correlated with audibility (*r* = 0.03 and –0.02, respectively), there was a modest correlation between dissimilarity and surprisal (*r* = 0.22), suggesting that both features captured some aspects of speech predictability. Nevertheless, the fact that the correlation was relatively low suggests that much of the variance in each of the two features captured distinct aspects of the linguistic content in the speech stimuli.

#### Feature-Specific Model Performance

After fitting the full multi-feature model as described above, we computed the unique contribution of each feature (except for word onsets; see below) to the overall model fit using procedures described in Broderick et al. (2021). Briefly, on each cross-validation fold, we estimated each feature’s contribution to the overall fit by comparing the goodness-of-fit for the full model to a null model, in which that feature’s contribution was eliminated. This was done by permuting regressor values of that feature, while maintaining their original timing. For all other features, the original regressors were used. Null model fits were computed by convolving the estimated TRFs with these regressors and correlating the predicted EEG waveform with the test data. This procedure was repeated 10 times to estimate the average null-model performance. Each feature’s model contribution was then computed as the difference between the goodness-of-fit metrics for the full model and its null model. Note that because feature values in the word onset regressor did not vary, the contribution of word onset to the overall model fit was zero by definition. As such, model fit contributions were only computed and analyzed for the remaining three features.

#### Regions of Interest

To strengthen our statistical analyses in light of inter-subject variability due to nuisance variables such as head shape and electrode cap placement, all analyses were performed on two regions of interest (ROI) derived by averaging model goodness-of-fit and TRFs from subsets of frontal and parietal electrodes (Figure 1B). The parietal ROI was chosen because of prior evidence that responses to higher-level features such as dissimilarity or surprisal tend to peak over parietal sites near electrode Pz (e.g., Broderick et al., 2018; Weissbart et al., 2019). The frontal ROI was included because we hypothesized that recruitment of frontal regions may aid prediction and disambiguation of the speech signals, particularly in challenging listening scenarios such as in the presence of a competing speaker.

#### Statistical Analysis

Group-level statistical analyses were applied to pooled outputs of single subject TRF analyses. Linear mixed-effects modeling (LMM), implemented using IBM SPSS 27, was used to assess how age group (YA vs. OA), ROI (frontal vs. posterior), model feature (dissimilarity, surprisal, and audibility), and attention (attended vs. ignored speech) related to feature-specific contributions to the TRF model’s goodness-of-fit. In addition to specifying the full interaction design in the fixed effects portion of the LMM, for each participant we included random intercept and random effects of feature and attention. We chose to include these random effects to reflect the possibilities that during speech processing high-level features may be weighed differently across participants, and that different participants may vary in their attentional control capabilities. Although this random effects specification was *a priori* reasonable, we verified that simpler and more complex specifications did not meaningfully change the outcome of the analysis. LMM was fit using maximum likelihood method, with Satterthwaite approximation for degrees of freedom, and diagonal covariance matrix pattern for random effects, in order to partially account for heteroscedasticity in the data. Goodness-of-fit values were scaled by 1000 in order to reduce the impact of rounding errors on the outcome of analysis. To statistically assess main effects and interactions, we used LMM ANOVA (type 3). Interactions were interpreted via *post hoc* examination of simple effects.

Comparisons of TRFs for the attended and ignored stories were performed for each TRF time point between 0 and 780 ms using two-tailed, paired-samples *t*-tests. Because this involved hundreds of statistical comparisons, we applied the *false discovery rate* (FDR; Benjamini and Hochberg, 1995) correction to control for the proportion of false positives among all significant discoveries. Similarly, between-group comparisons (i.e., YA vs. OA) were performed on TRF time courses, with two-sample *t*-tests applied separately to the attended and ignored TRFs and corrected using the FDR method.

As a potentially more sensitive alternative to TRF analyses utilizing pre-determined ROIs, we also conducted cluster-based permutation analyses (Maris and Oostenveld, 2007) utilizing all 64 electrodes. Briefly, this non-parametric approach assesses differences between two conditions by comparing spatio-temporal clusters of a particular test statistic (e.g., the sum of spatially and temporally adjacent t-statistics that exceed a given uncorrected α threshold) to an empirically computed null distribution for this statistic. The null distribution is established by repeated random permutation of the labels for the two conditions (i.e., assuming that there is no difference between the conditions), each time splitting the data according to the permuted labels and computing the same cluster statistics. The maximum-valued cluster statistic from each iteration is used to build the null distribution. Statistical significance for each of the clusters present in the original data is then established by computing the probability of exceeding that test statistic value within the null distribution. In practice, we utilized the *ft_timelockstatistics* function from FieldTrip toolbox (Oostenveld et al., 2011) for MATLAB (configuration parameters: ‘montecarlo’ method for computing cluster statistics, ‘indepsamplesT’ statistic for forming clusters when assessing effects of attention, with α = 0.05, ‘depsamplesT’ statistic for effects of age, ‘maxsum’ cluster statistic, and 1000 iterations for establishing the null distribution).

Finally, exploratory correlation analyses were performed on different combinations of neural (e.g., full model goodness-of-fit, feature-wise model contributions, TRF amplitudes) and behavioral (e.g., comprehension, confidence, and SSQ_m_ scores) metrics. In these analyses, we corrected each set of correlation statistics using the Bonferroni correction. Importantly, this represents less stringent multiple comparisons correction than correcting by the total number of comparisons across all combinations of correlation analyses. This choice was made as an attempt to improve the balance between the likelihood of Type I and Type II errors in these exploratory analyses.

## Results

### Behavioral Measures of Speech Understanding

Following each 1-min block of listening to a two-talker speech mixture, participants responded to four true/false questions about the content of the attended story and indicated confidence about their response. The average performance on this comprehension task was 83% (*SD*: 7.5%, 64.4 – 94.2% range), significantly above the 50% chance level [*t*(40) = 28.4, *p* < 0.001], indicating that participants were successfully able to attend to the target speaker and comprehend the content of the story. At the same time, the fact that these scores were considerably below ceiling is an indication that the two-talker mixture produced overall a challenging listening scenario. Older participants showed a trend of performing better than younger participants (YA: mean ± SD = 80.8 ± 7.9%, OA: 85.1% ± 6.5%), but this difference did not reach statistical significance (*z* = –1.8, *p* = 0.07, Mann–Whitney *U*-test). When treating age as a continuous variable, its association with the proportion of correct responses was also non-significant (*r* = 0.14, *p* = 0.4). Confidence measures showed the same general pattern of results as the comprehension scores and the two measures were positively correlated [*r* = 0.68, *p* < 0.001], indicating that participants had good awareness of their performance.

Because hearing loss was more common among the older participants, and we compensated for it by amplifying the audio in frequency ranges of elevated thresholds (see section “Materials and Methods”), we assessed whether this amplification could account for the difference in performance. As expected, in the portion of participants who received amplification (*n* = 18), there was no relationship between average high-frequency audiogram (2–8 kHz range), and comprehension-performance (*r* = –0.23, *p* = 0.37) or confidence (*r* = 0.12, *p* = 0.63) scores. The same pattern was observed when using the average of the entire 0.25–8 kHz range of audiometry. As such, there was no evidence that amplification had an impact on performance, or that it could account for the marginal difference in the between-group comparison of performance.

Prior to the experimental session, each participant filled out a modified subset of the SSQ (SSQ_m_) questionnaire to assess their subjective difficulties with SIN perception. We found no significant difference in these measures between younger and older participants (*z* = –0.37, *p* = 0.71, Mann–Whitney *U*-test), and no correlation between SSQ_m_ score and the proportion of correct responses from the behavioral task (*r* = –0.13, *p* = 0.43), or between SSQ_m_ and high-frequency hearing loss (*r* = –0.03, *p* = 0.92).

### Cortical Measures of Speech-Mixture Processing

In order to characterize cortical responses to semantic content of speech, we applied computational models to EEG responses measured while participants listened to a mixture of two distinct narrative stories, while attending to one of them. The features included in the model were word onsets, word audibility reflecting word-by-word fidelity of the incoming acoustic signal, semantic dissimilarity reflecting short-term (sentence timescale) dissimilarities between the word2vec vector characterizing each word and its immediately preceding context, and word surprisal reflecting long-term predictability of each word given the preceding multi-sentence context.

**FIGURE 2:**
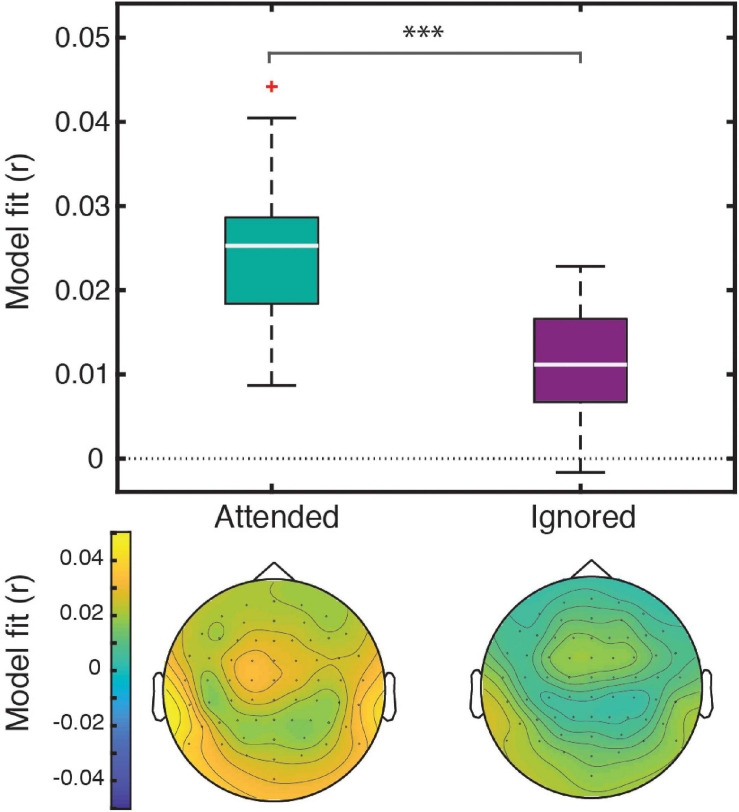
The four-feature model explained a significant amount of variance in responses to both attended and ignored speech. Box plots **(top)** represent distributions of goodness-of-fit values averaged over electrodes across all participants. The significance level (***) for the statistical comparison between attended and ignored speech corresponds to *p* < 0.001. The topographic plots **(bottom)** depict the distribution of goodness-of-fit values for attended and ignored speech across the scalp.

**FIGURE 3:**
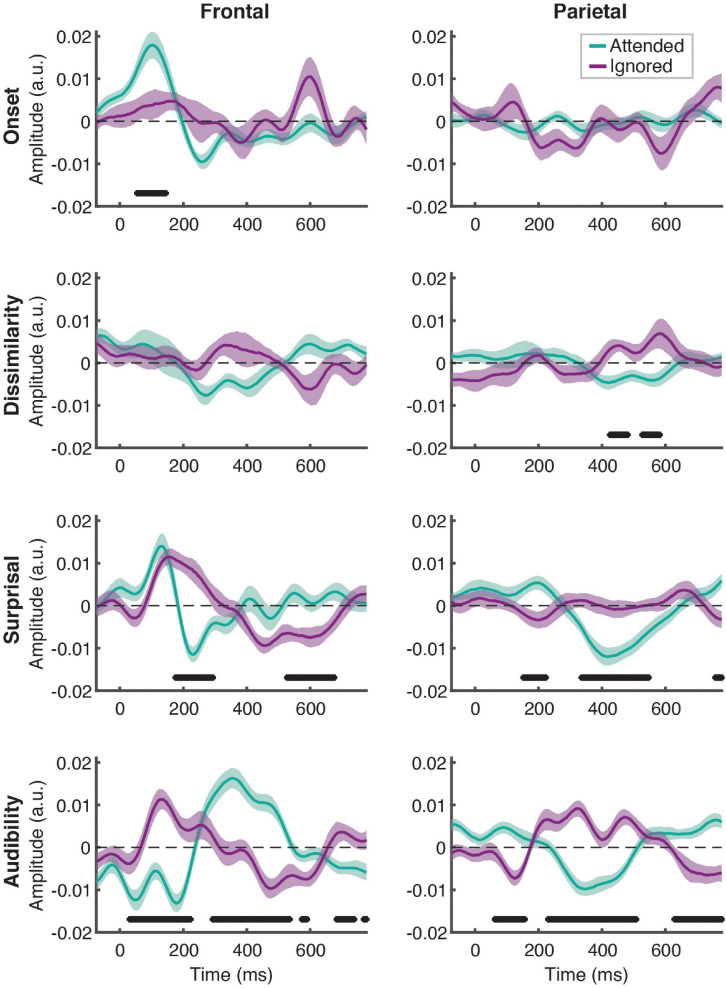
Attentional modulation of feature-specific responses. Each plot depicts the comparison of TRFs averaged across all participants for attended (green) and ignored (purple) speech for each of the features (panel rows) and ROIs (panel columns). The upper and lower bound of each curve represents ± 1 standard error (SE) of the mean. Black horizontal bars at the bottom of the plots indicate time intervals over which attended and ignored TRFs differed significantly at the FDR-corrected level, with α = 0.05.

Linear regression of these features against the EEG signal produced responses that explained a significant amount of variance in the data pooled across participant groups and electrodes, as reflected by a significant positive correlation between the full-model EEG prediction and held-out data for both attended [*t*(40) = 20.95, *p* < 0.001] and ignored [*t*(40) = 13.47, *p* < 0.001] speech, with a significantly stronger fit for the former [*t*(40) = 9.07, *p* < 0.001; Figure 2]. The same pattern of results was observed when examining model fits in frontal and parietal ROIs. Figure 3 depicts the average attended (green) and ignored (purple) TRFs in the two ROIs for each of the features included in the model. Attended speech elicited robust neural responses for most of the features included in the model, with prominent early (∼100 ms) peaks observed frontally for onsets and surprisal, and late (∼400 ms) peaks observed frontally for audibility, and parietally for surprisal and audibility. In contrast, ignored speech elicited flatter and noisier responses, with prominent peaks only appearing for surprisal and audibility. Direct comparison of attended and ignored TRFs revealed significant attentional modulation for all features, as depicted by black horizontal bars at the bottom of each TRF plot (indicating time points corresponding to FDR-corrected significant effects of attention). This modulation was most pronounced for surprisal and audibility, which exhibited differences at both earlier (∼200 ms) and later (400 and 600+ ms) time points in at least one of the ROIs. Consistent with these results, supplementary cluster-based permutation test analyses also indicated that attended and ignored speech produced significantly different responses for each of the four features (see Supplementary Figure 1).

**FIGURE 4:**
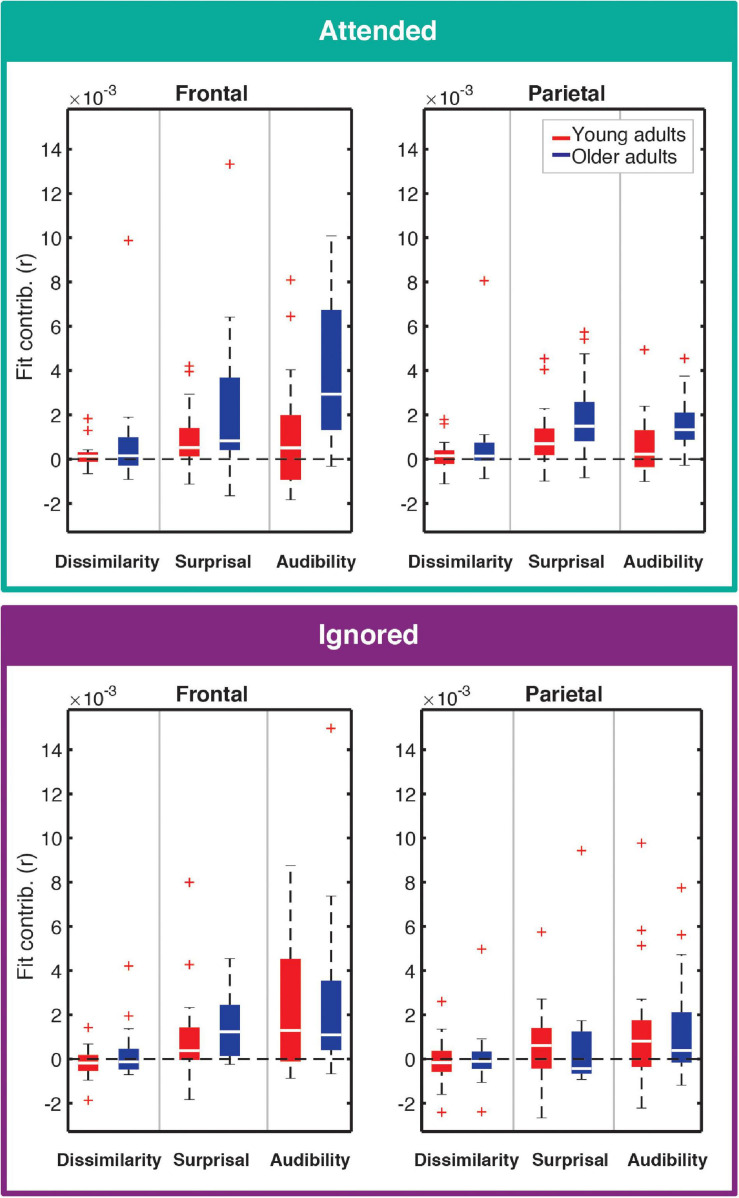
Feature-specific contributions to the model fit for attended **(top)** and ignored **(bottom)** responses. Each panel depicts the box plot of model fit contributions for each of the three features in the younger (red) and older (blue) adult groups. Left and right panels represent results for frontal and parietal ROIs, respectively. Goodness-of-fit contributions for the word-onset feature were by definition 0 due to the permutation-based approach to compute these contributions, and are therefore not included in the figure. Note that some points are depicted with red + signs as outliers in order to better depict where the bulk of the data points lie within the fit contribution distributions. However, all data points were utilized in statistical analyses described in the text.

Contributions of each feature (except for onsets; see section “Feature-Specific Model Performance”) to the overall model fit for both age groups are plotted in Figure 4. Model fit contribution values represent the difference in goodness-of-fit for the held-out EEG data between the full model and null models in which a given feature’s regressor was selectively disrupted by shuffling its feature amplitudes (see section “Feature-Specific Model Performance”). Thus, for a particular feature, a model fit contribution exceeding 0 represents the scenario where the EEG responses scaled, to some degree, with that feature’s regressor values. To compare how these model contributions differed in the two age groups, we performed a LMM ANOVA with within-subject factors of ROI, model feature, and attention, and a between-subjects factor of age group (Table 1). We found a main effect of age group [*F*(1,41.6) = 6.46, *p* = 0.015], reflecting stronger overall goodness-of-fit contributions in older than younger adults. We also found a main effect of feature [*F*(2,60) = 24.05, *p* < 0.001], stemming from stronger tracking of surprisal and audibility than dissimilarity (both *p* < 0.001), and a trend of stronger tracking of audibility than surprisal (*p* = 0.03, with α = 0.017). Finally, we found a main effect of ROI [*F*(1,358.7) = 14.12, *p* < 0.001], reflecting stronger goodness-of-fit contributions in the frontal than parietal ROI. Surprisingly, despite clear attentional modulation in both the overall goodness-of-fit and the TRFs (i.e., Figures 2, 3), the main effect of attention was non-significant [*F*(1,41) = 3.01, *p* = 0.09], reflecting only a trend of stronger tracking of attended features.

**TABLE 1:** LMM ANOVA results.

	df	*F*
Age	1, 41.6	6.46*
Feature	2, 60	24.045***
Attend	1, 41	3.01
ROI	1, 358.7	14.115***
Age × Feature	2, 60	0.694
Age × Attend	1, 41	5.186*
Age × ROI	1, 358.7	4.121*
Feature × Attend	2, 358.7	1.155
Feature × ROI	2, 358.7	5.815**
Attend × ROI	1, 358.7	0.022
Age × Feature × Attend	2, 358.7	2.024
Age × Feature × ROI	2, 358.7	0.693
Age × Attend × ROI	1, 358.7	0.593
Feature × Attend × ROI	2, 358.7	0.952
Age × Feature × Attend × ROI	2, 358.7	0.775

Significant *F*-statistic values are bold, with levels of significance: *p < 0.05, **p < 0.01, ***p < 0.001.

In addition to these main effects, we detected several significant interactions. There was a significant interaction between attention and age group [*F*(1,41) = 5.19, *p* = 0.028], reflecting an overall greater difference between attended and ignored fits in older than younger participants [YA: *F*(1,41) = 0.14, *p* = 0.71; OA: *F*(1,41) = 8.25, *p* = 0.006]. A significant interaction between ROI and age group [*F*(1, 358.7) = 4.12, *p* = 0.043] was associated with stronger contributions to model fits across features at the frontal compared to the parietal ROI in older adults [YA: *F*(1,358.7) = 1.46, *p* = 0.23; OA: *F*(1,358.7) = 17.16, *p* < 0.001]. Finally, we found a significant interaction between feature and ROI [*F*(2,358.7) = 5.82, *p* = 0.003], reflecting stronger differential in tracking between frontal and parietal ROIs for audibility than for dissimilarity and surprisal [Dissimilarity: *F*(1,358.7) = 0.05, *p* = 0.83; Surprisal: *F*(1,358.7) = 2.03, *p* = 0.16; Audibility: *F*(1,358.7) = 23.67, *p* < 0.001]. While our statistical analyses were ROI-based, for illustrative purposes we also provide topographies of goodness-of-fit differences between age groups for each of the features (see Supplementary Figure 2).

Although the goodness-of-fit analyses above indicate that there are significant differences in processing of attended and ignored speech between younger and older participants, they do not provide insight into the timing and amplitude of the underlying neural responses. To explore if our data contain evidence of age-related differences in neural responses, we statistically compared TRF amplitudes between the two age groups at each time point in the 0–780 ms range. Because these analyses involved hundreds of point-by-point comparisons between groups, we applied FDR correction, and focused on comparisons at the level of individual features, rather than utilizing more complex interaction metrics. As such, these analyses were relatively rudimentary, and should be considered as exploratory in nature. As an alternative, and potentially more sensitive approach to detecting group differences, we also compared group TRFs across the entire scalp using cluster-based permutation analyses (Maris and Oostenveld, 2007). Note that in their implementation here, these latter analyses test for group differences without statistical claims about the timing or spatial localization of these differences (Sassenhagen and Draschkow, 2019).

**FIGURE 5:**
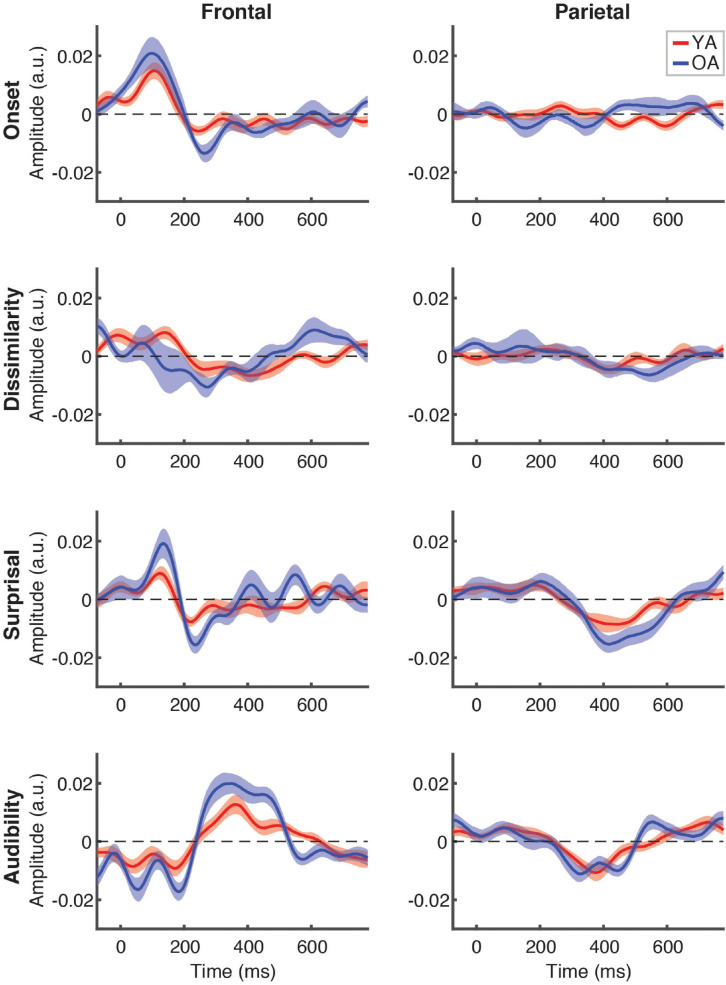
Between-group comparison of TRFs for attended speech. Each plot depicts a comparison of TRFs between younger (red curves) and older (blue curves) participants, for different features **(panel rows)** and ROIs **(panel columns)**. Note that the TRF amplitudes did not differ significantly between groups at the FDR-corrected level at any time point for any of the features (but see Supplementary Figure 3).

Figure 5 depicts the differences in responses to the attended speech between younger (red lines) and older (blue lines) participants, separately for each feature (plot rows) and ROI (plot columns). Across the features, we did not detect any significantly different time points at the FDR-corrected level, although there was a trend for OA to show higher-amplitude TRFs, as indicated by greater TRF deflections from 0 for the OA than YA. This trend was most pronounced for audibility at the frontal ROI between ∼250 and 500 ms, where TRF values for a range of time points differed between groups at the uncorrected level (not pictured). Supplementary cluster-based permutation analyses detected significantly stronger response to audibility by OA (*p* = 0.005, with α = 0.006) with the cluster underlying this difference including central electrodes around at the ∼300–450 ms latency (see Supplementary Figure 3).

**FIGURE 6:**
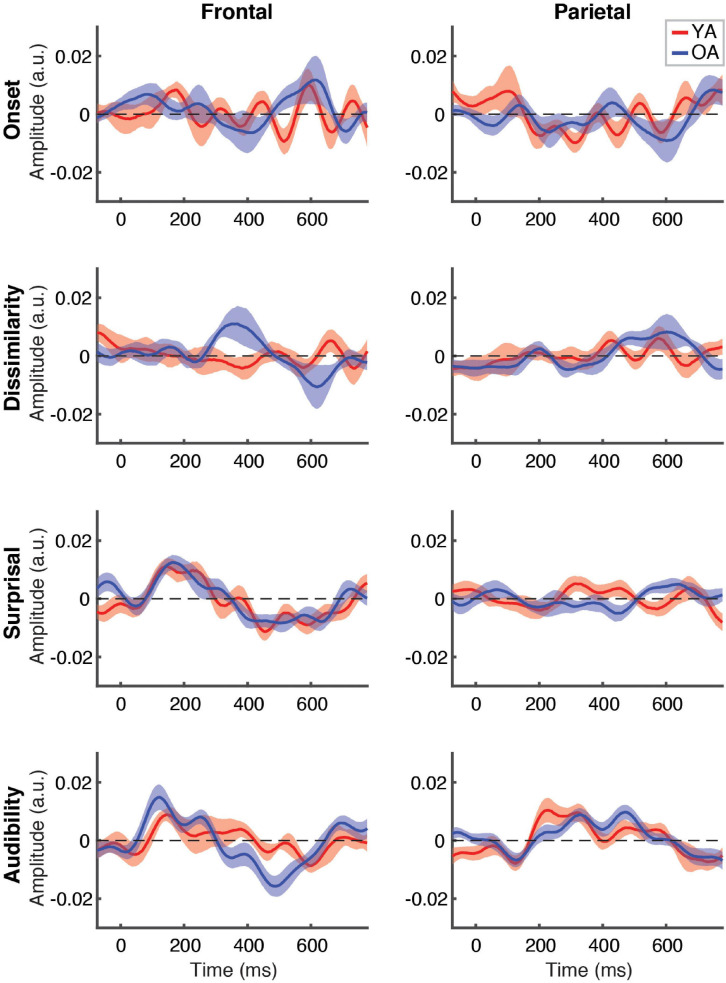
Between-group comparison of TRFs for ignored speech. Subplot arrangement and statistical comparisons are as in Figure 5. Note that the TRF amplitudes did not differ significantly between groups at the FDR-corrected level at any time point for any of the features (but see Supplementary Figure 4).

Between-group comparison of TRFs for ignored speech are shown in Figure 6. Unlike responses to attended speech, most features, with the exception of frontal TRFs for surprisal and audibility, showed largely flat response patterns that did not differ between the groups. As with attended speech, the frontal audibility TRF showed the most pronounced trend towards a group difference at around the ∼500 ms latency, with the OA TRF showing a greater negative deflection compared to YA. Cluster-based permutation test detected a significantly more negative response for audibility in the OA group (*p* = 0.006, α = 0.006), with the cluster underlying this difference including fronto-central electrodes between ∼400 and 550 ms (see Supplementary Figure 4).

To complement the exploratory point-by-point and cluster-based analyses, we also conducted between-groups analyses specifically targeted at comparing responses in the time range of the N400 response. To this end, we compared each feature’s average TRF amplitudes in the 300–500 ms range. Because previous work found little to no evidence of N400 for ignored speech, these comparisons were only done for attended speech. This analysis revealed that YA had significantly weaker frontal response to audibility than OA [*z* = –2.96, *p* = 0.003, α = 0.006, given the total number of eight comparisons), consistent with the trends seen in Figure 5. No other feature approached significant difference in either ROI.

### Neuro-Behavioral Correlations

We next sought to examine how our electrophysiological measures related to behavioral responses during the experiment, and the SSQ_m_ scores obtained prior to this experiment. To this end, we conducted a number of exploratory analyses, including correlations between behavioral measures and the overall model goodness-of-fit, feature-specific model contributions, and the average TRF amplitudes in the 300–500 ms time range. Given the number of these analyses, and our limited sample size, we focused our analyses on full participant samples, rather than age group comparisons. Because of the less stringent multiple comparisons correction procedure (only correcting by the number of statistical tests within each analysis), significant effects in this section should be interpreted as trends rather than robust statistical effects.

**FIGURE 7:**
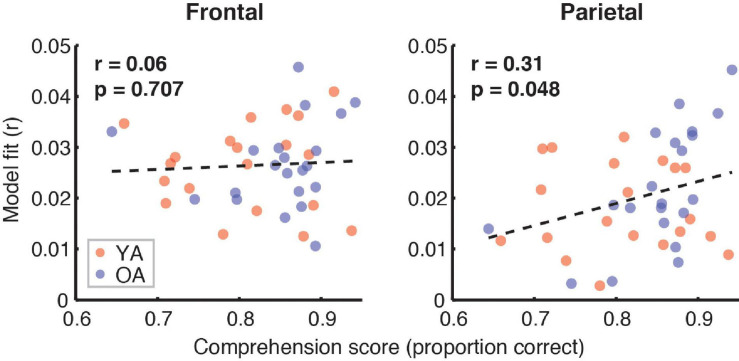
Scatterplots showing the relationship between the full model goodness-of-fit and the proportion of correct responses on the comprehension questions. Symbols represent data from individual participants pooled across the two age groups, YA (red symbols) and OA (blue symbols). Pearson’s correlation coefficients and the corresponding uncorrected *p*-values, with α = 0.025, are shown for frontal (left plot) and parietal (right plot) ROIs. Due to the exploratory nature of this analysis, relationships present in the data should be interpreted as trends.

**FIGURE 8:**
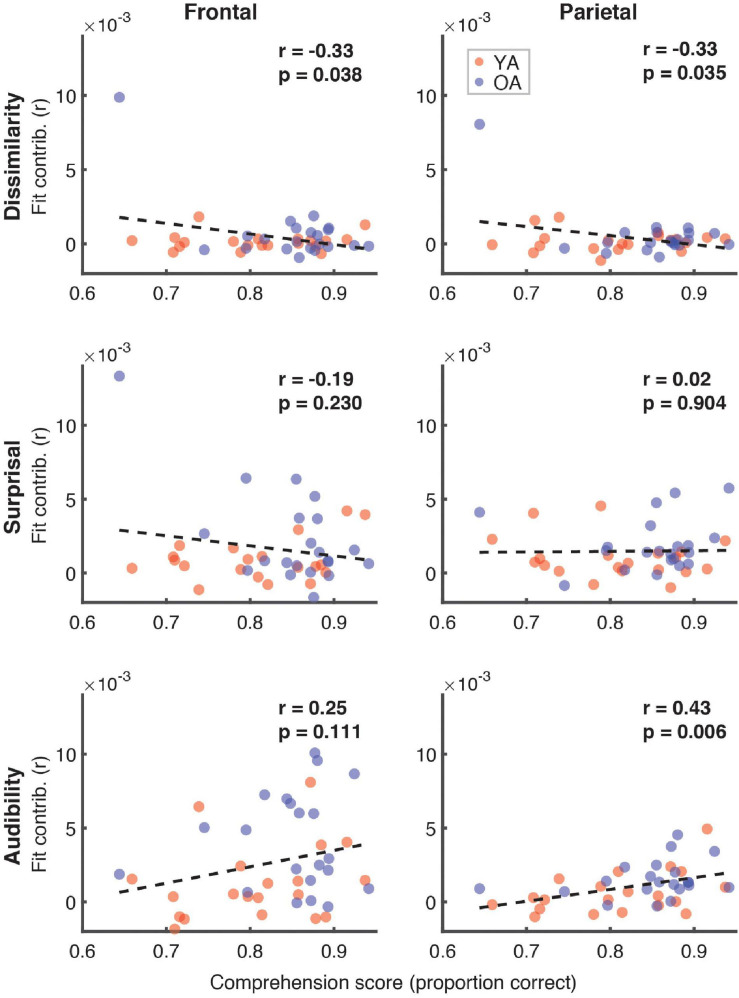
Scatterplots of comprehension scores and feature-specific model contributions. Different rows of panels refer to different features and different columns correspond to the two ROIs. Red and blue symbols represent data from YA and OA groups, respectively. Pearson’s correlations and the corresponding uncorrected *p*-values, with α = 0.008, are shown in the upper portion of each panel. Due to the exploratory nature of this analysis, relationships present in the data should be interpreted as trends.

Figure 7 depicts the relationship between the proportion of correct responses on comprehension questions during the experiment, and the overall model goodness-of-fit in the frontal (left panel) and parietal (right panel) ROIs. Red and blue symbols depict data from YA and OA participants, respectively. While we observed no relationship in frontal regions (*r* = 0.06, *p* = 0.71), there was a non-significant positive association trend between the two measures (*r* = 0.31, *p* = 0.048, Bonferroni-corrected α = 0.025) in the parietal ROI. A similar pattern of results was observed when proportion of high-confidence responses for the comprehension questions was used instead of the performance itself. Relationships between the proportion of correct responses and feature-specific contributions to the model fit are depicted in Figure 8. We observed a trend for a positive association for word audibility in the parietal ROI (*r* = 0.43, *p* = 0.006, α = 0.008). Although dissimilarity showed a negative trend in both ROIs, this result was driven by the single outlier data point from the OA group.

**FIGURE 9:**
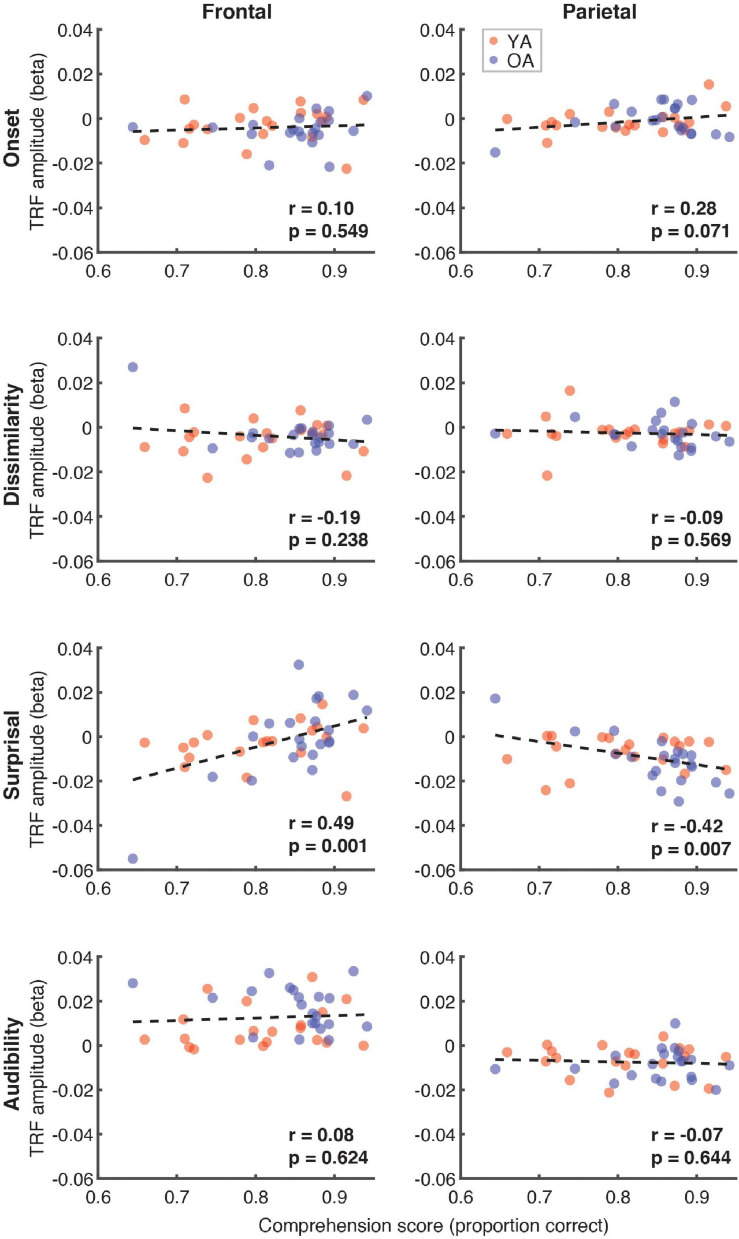
Scatterplots of comprehension scores and mean TRF amplitudes between 300 and 500 ms. Figure layout and color-coding of data points is as in Figure 8. The depicted *p*-values are uncorrected, with α = 0.006. Due to the exploratory nature of this analysis, relationships present in the data should be interpreted as trends.

Next, we explored the possible relationship between the comprehension scores (proportion correct) and the average TRF amplitude in the 300–500 ms time range, when N400 effects generally appear parietally. These analyses, shown in Figure 9, revealed a trend towards a positive relationship for surprisal in frontal (*r* = 0.49, *p* = 0.001) and a negative relationship in parietal (*r* = –0.42, *p* = 0.007, α = 0.006) regions. Although this analysis focused broadly on the time range of N400, the frontal trend was associated with positive, rather than negative deflections in the TRF.

**FIGURE 10:**
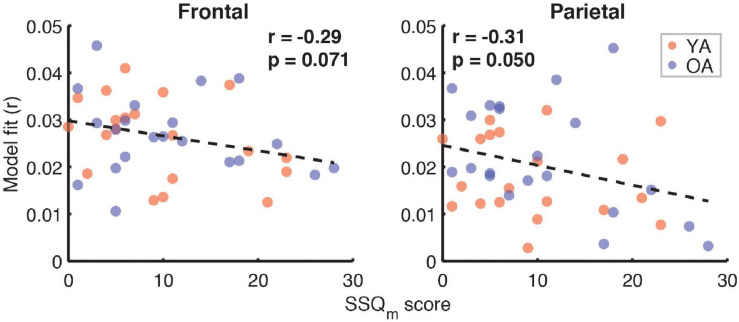
Scatterplots of SSQ_m_ scores and overall model goodness-of-fit for frontal **(left panel)** and parietal **(right panel)** ROIs. Note that a higher score on SSQ_m_ questionnaire reflects a greater difficulty with understanding speech in noise. The depicted *p*-values are uncorrected, with α = 0.025. Due to the exploratory nature of this analysis, relationships present in the data should be interpreted as trends.

Correlation analyses examining the relationship between subjective SIN perception difficulties, captured by the SSQ_m_ scores, and the full model goodness-of-fit metric (Figure 10) revealed trends toward a negative relationship in both the frontal (*r* = –0.29, *p* = 0.07) and parietal ROIs (*r* = –0.31, *p* = 0.05, α = 0.025). However, analyses of relationships with feature-specific TRF amplitudes and model contributions revealed no feature for which these trends were apparent.

**FIGURE 11:**
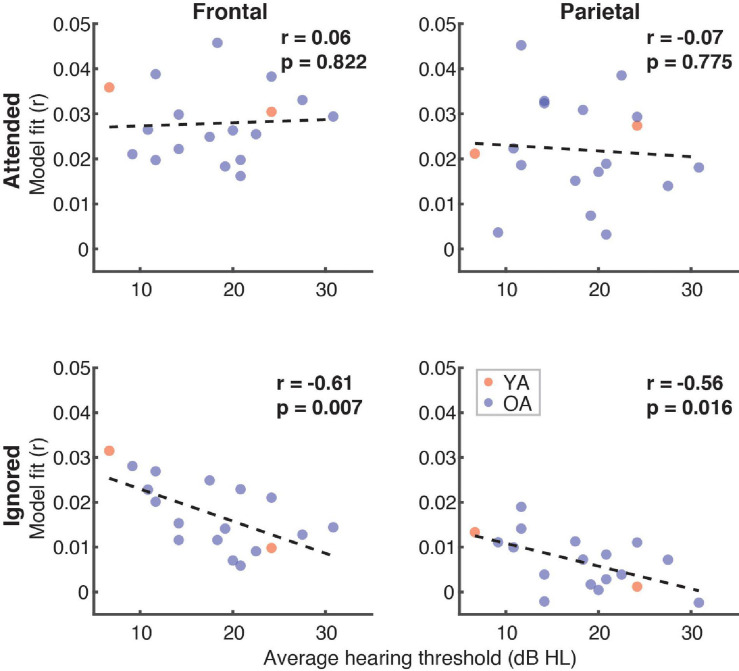
Scatterplots of average high-frequency hearing thresholds (2–8 kHz) in participants with hearing loss and overall model goodness-of-fit as a function of attention **(panel rows)** and ROI **(panel columns)**. The depicted *p*-values are uncorrected, with α = 0.013. Due to the exploratory nature of this analysis, relationships present in the data should be interpreted as trends.

Finally, because a portion of the participants had mild hearing loss at high frequencies (which was compensated for by amplifying speech in the corresponding frequency ranges; see section “Materials and Methods”), we examined if and how high-frequency (2–8 kHz) hearing thresholds in these participants related to the overall model fits (Figure 11). Although we found no relationship between the average hearing thresholds over the 2–8 kHz range and model goodness-of-fit for attended speech (Frontal ROI: *r* = 0.06, *p* = 0.82; Parietal ROI: *r* = –0.07, *p* = 0.76), there was a significant negative correlation for ignored speech frontally (*r* = –0.61, *p* = 0.007) and a negative trend parietally (*r* = –0.56, *p* = 0.016, α = 0.013). At the level of feature-specific contributions to the model fit, we observed a trend of a negative correlation with dissimilarity (*r* = –0.49, *p* = 0.04, α = 0.008). However, because this feature had overall near-zero fit contributions, this trend was likely spurious.

## Discussion

Existing objective (laboratory and clinical) measures of speech perception have shown surprisingly poor correlations with self-reported difficulties with speech recognition in noise that arise from aging and/or hearing impairment (Phatak et al., 2018; Smith et al., 2019). In the present study, we measured EEG responses to continuous two-talker speech mixtures in younger (< 40 y.o.) and older (> 40 y.o.) participants to investigate changes in neural responses to masked speech with increasing age. Participants’ cortical responses were predicted by modeling TRFs for four speech features: word onsets, semantic dissimilarity, lexical surprisal, and word-level audibility. We also collected behavioral measures, including participants’ subjective ratings of their difficulties with SIN understanding (modified SSQ), and comprehension scores for attended speech during the experiment and the associated confidence ratings.

Two of the features, surprisal and audibility, emerged as the model’s main contributors to the variance in the EEG data, suggesting that these features captured stimulus characteristics actively tracked by our participants’ auditory systems. The contributions were particularly evident for attended speech (Figure 4). In addition to robust tracking of word-level features, we found that participants’ performance on the comprehension task (Figure 7) and the associated confidence ratings showed a trend towards a positive correlation with the overall model goodness-of-fit for the attended speech. This suggests that successful tracking of word-level features is associated with improved speech comprehension. Due to the exploratory nature of our neuro-behavioral analyses, we were unable to establish robust statistical links between performance and model contributions, or TRF magnitudes, for any one of the model features. However, we did find trends towards an association between performance and audibility model fit contributions in the parietal ROI, and between performance and surprisal TRF amplitudes in both ROIs. Speculatively, these trends suggest that improved comprehension may be related to at least two cognitive processes. First, the association with audibility suggests that improved performance may stem from more effective weighing of word-level information by word reliability, as reflected by the word SNR. Second, the association with surprisal suggests that high performance may be related to increased sensitivity to lexical and/or semantic associations between different segments of speech.

Consistent with previous work on neural representations of two-talker speech (Ding and Simon, 2012a; Mesgarani and Chang, 2012; Broderick et al., 2018; O’Sullivan et al., 2019) we found that neural responses to a speech mixture preferentially reflect attended speech, while representations of distractor speech are comparatively suppressed. This attentional modulation was more apparent in TRFs than model-fit contributions.

Comparisons of EEG responses between age groups revealed that OAs exhibited on average greater differences in feature-specific model-fit contributions between attended and ignored speech than YAs. This age effect was driven primarily by stronger fits for attended speech in the frontal ROI (see Figure 4). Although to a weaker degree, these differences were mirrored in attended TRFs, in that OAs showed generally stronger TRF deflections from 0 compared to YAs (Figure 5). However, these TRF differences did not reach statistical significance when controlling for false discovery rate, and we only detected group differences for audibility (both attended and ignored) using cluster-based permutation methods. The discrepancy between goodness-of-fit and TRF metrics may have stemmed from nuisance factors such as inter-subject variability in cortical geometry, and/or inadequate sample size.

It is notable that the comprehension scores of OAs tended to be higher than that of YAs, despite greater prevalence of hearing loss (16 out of 18 participants in the OA group had some degree of HI). This finding complicates the interpretation of age-related differences in neural responses. It may be the case that OAs in our participant sample were either more engaged, or exerted greater effort in the task, which in turn led to stronger speech tracking in their EEG data, as well as marginally better performance. As such, an important direction for future work will be to examine how cognitive factors such as task engagement and effort relate to electrophysiological measures of speech processing.

### Relationship to Existing Work on Age-Effects on Electrophysiological Measures of Speech Processing

Several studies have examined effects of age (Presacco et al., 2016; Decruy et al., 2019; Zan et al., 2020) and hearing loss (Millman et al., 2017; Decruy et al., 2020) on continuous speech processing in the context of envelope tracking. Generally, these studies have demonstrated that older adults and those with hearing loss exhibit exaggerated cortical tracking of speech envelope both in quiet and in the presence of a competing speaker. Our analyses show a similar pattern of amplified feature tracking in the aging population, albeit for word-level features. Responses to the audibility feature, in particular, may reflect similar underlying processes as those involved in envelope processing. However, audibility in our study was defined as the word-by-word ratio between the acoustic energy in the two speech waveforms, rather than the absolute amplitude of each speech signal, making direct comparisons of the two measures difficult. Distinct from envelope TRFs, the audibility TRF in our study contained prolonged deflections from 0 in the 300–500 ms latency range, suggesting that our measure may tap into additional higher-level processes (notably, word-onset TRFs only contained robust response at latencies prior to ∼250 ms). Although high-level features such as lexical surprisal are seemingly unrelated to lower-level features such as speech envelope, it is possible that predictive processes may interact with lower-level stimulus encoding via feedback processes, as has been demonstrated for dissimilarity (Broderick et al., 2019).

While measures of envelope tracking have provided important insights into speech processing, they are largely uninformative about the nature of higher-level processes involved in speech perception. In recent years, an increasing number of studies have investigated the relationship of electrophysiologically measured cortical responses to phoneme- and word-level representations related to lexical processing (e.g., Brodbeck et al., 2018), as well as syntactic and semantic (Broderick et al., 2018; Heilbron et al., 2019; Weissbart et al., 2019; Donhauser and Baillet, 2020) processing. Nevertheless, relatively little is known about how these representations change as a function of age, particularly in challenging listening conditions. Recently, Broderick et al. (2021) compared representations of semantic dissimilarity and 5-gram lexical surprisal derived from responses to clean speech in younger and older adults. They showed that although younger adults exhibited robust responses to each feature, older adults only showed strong responses to lexical surprisal (albeit with a delayed peak response), with a nearly absent response to semantic dissimilarity. These results were interpreted as potentially reflecting lesser reliance of older adults on semantic predictive process due to age-related cognitive decline. Consistent with this, older participants with greater semantic verbal fluency, a measure related to the ability to engage in semantic prediction, showed greater contribution of semantic dissimilarity to the model of cortical responses to speech.

Because our experimental design involved listening to a more challenging, two-speaker mixture, direct comparisons of our results with those of Broderick et al. (2021) are not possible. Nevertheless, there are marked differences between the patterns of results observed in their study compared to ours. In particular, we observed overall stronger feature tracking in older than younger adults, particularly at the frontal ROI. However, the general pattern of relative strength of tracking across features did not differ between groups.

In Broderick et al. (2021), the greatest age-related differences were shown for semantic dissimilarity, whereas our goodness-of-fit results showed relatively weak contributions from this feature (compared to surprisal and word audibility) that did not differ significantly between the younger and older age groups. Additionally, we did not observe robust dissimilarity-related N400 response in either group, in contrast to the significant parietal N400 in the TRF for dissimilarity in older but not younger adults reported by Broderick et al. (2021). Although this discrepancy is puzzling given the use of nearly identical methods for computing dissimilarity, one contributing factor to this may have been that our study used different referencing procedures (common average vs. mastoid reference in Broderick et al., 2021), which could affect the amplitudes and topography of the N400 responses. Nevertheless, because we did observe robust parietal N400-like responses for surprisal and audibility, it seems unlikely that the referencing strategy prevented the observation of the N400 response for dissimilarity. Another possible reason for the discrepancy is that our regressors included feature values for both content and function words, while Broderick et al. (2021) only analyzed responses to content words. However, re-analysis of our data utilizing just content word regressors (not shown here) resulted in TRFs and model fit patterns that were highly similar to all-word regressors, albeit slightly noisier. Therefore, it may be that the utility of dissimilarity is limited if other features, which better capture neural responses that would otherwise be attributed to dissimilarity, are included in the model.

Another important difference between the two studies pertains to the role of surprisal in the models fitted to the data. Specifically, unlike the relatively simple 5-gram surprisal used by Broderick et al. (2021), the surprisal features utilized in our study were computed using an advanced natural language model (GPT-2; Radford et al., 2019) that uses preceding context of up to several hundred words (i.e., dozens of sentences) in order to estimate each upcoming word. As such, surprisal in our study likely captured responses related to higher-level lexical and/or syntactic predictions. Thus, although responses to these two surprisal measures cannot be directly compared, the robust tracking of surprisal by younger and older adults in our study is consistent with reliance on predictive processes in both of these populations.

Importantly, the seemingly conflicting pattern of results between these studies could in fact reflect two distinct contributors to speech perception difficulties in older adults, namely decreases in the fidelity of lower level representations, and cognitive decline. Prevalence of mild high-frequency hearing loss in our sample of older adults was quite high, making it likely that decreased fidelity of peripheral representations had an effect on our results. While Broderick et al. (2021) did not report audiogram measures for their sample of older adults, the mean age was considerably greater in their study (mean ± SD = 63.9 ± 6.7 years vs. 53.5 ± 8.7 years in this study), making it likely that similar or greater hearing difficulties may have impacted their participants. However, because of the age difference in the two samples, the effects of cognitive decline may have contributed more significantly to the results of Broderick et al. (2021), and may potentially explain why measures related to predictive processes showed different effects in the two studies.

### Possible Mechanisms of Age-Related Amplification of Speech Tracking

The present study represents one of the initial attempts to characterize and compare responses to high-level features in two-talker speech mixtures from younger and older adults. As such, one of our goals was to broadly explore these responses and their relationship to behavioral measures, in order to provide a relatively rich reference point for future work on this topic. However, inclusion of relatively extensive exploratory analyses came at the cost of lower power for detecting statistical effects. Additionally our study design notably did not involve direct experimental manipulation of any of the speech features, but instead exploited the natural variation of these features in the speech materials.

While we are cautious about making mechanistic interpretations of our results given the above reasoning, we speculate that there are at least two underlying factors that may have given rise to the pattern of amplified feature tracking in the OA group. First, because the OAs showed a trend of better comprehension performance than the YAs, it appears plausible that the two groups differed in their utilization of cognitive resources related to executive function. More specifically, it may be the case that the older group engaged in the task more effortfully, leading to both stronger feature tracking, and marginally increased performance. Past behavioral work has demonstrated that listening effort, as quantified using pupillometry, is monotonically related to spectral resolution of speech (Winn et al., 2015), such that comprehension of lower-fidelity speech requires greater listening effort even when performance is at ceiling. Since mild hearing loss was more prevalent in our OA group, it may be the case that lower-fidelity in their peripheral speech representation required exertion of greater effort than that of the YA group. Indeed, pupillometry data from older adults with mild hearing loss have demonstrated that this group exerts greater listening effort than younger normal hearing population, even in a simple word identification task (Ayasse et al., 2017).

Another possible factor that may have subserved the age differences in feature tracking in our study is the degree of utilization of contextual cues during comprehension. Specifically, a key compensatory mechanism for the poorer fidelity of peripheral representation in the aging population may be increased reliance on the successfully identified segments of speech to aid inference about speech segments with lower SNR. Indeed, greater dependence on contextual cues for speech comprehension has been demonstrated in populations with compromised representations of speech, including those with hearing loss (Benichov et al., 2012; Lash et al., 2013) and cochlear implants (Amichetti et al., 2018; Dingemanse and Goedegebure, 2019; O’Neill et al., 2019). In the context of our feature-tracking model, a possible manifestation of heightened reliance on predictive processes would be increased tracking of surprisal. Although we did not find evidence that surprisal, specifically, was tracked differentially by the two age groups (i.e., there was no interaction between age and feature), the overall greater model contributions in the OAs is broadly consistent with the possibility that predictive processes were more actively engaged in that group. Additionally, because audibility-related responses showed prolonged peaks at relatively late latencies (e.g., see peaks at 250–550 ms in Figure 3), we speculate that the tracking of this feature may also reflect utilization of more intelligible speech segments in higher-level processes, such as prospective prediction and retrospective disambiguation and/or error-correction (Rönnberg et al., 2019, 2021).

An important caveat with respect to the two factors discussed above is that the hypothesized increased use of contextual cues by older adults is unlikely to be independent from increased listening effort. In fact, listening effort is thought to reflect the combined effect of an array of cognitive processes involved in attention and working memory (see review by Peelle, 2017). As such, increased reliance on predictive processes may be just one of many manifestations of increased listening effort.

### Higher-Level Speech Feature Tracking as an Index of Speech in Noise Perception Difficulties

A key reason for our choice to study responses to lexical and semantic features is their potentially greater sensitivity to SIN perception difficulties, compared to responses driven by lower-level features such as the speech envelope. Because dissimilarity and surprisal (but not audibility) depend on preceding lexical and semantic context, in order for language processing mechanisms to accurately track them, each word within the sequence needs to be recognized and integrated with the preceding context. Lower-level SIN processing deficits may thus disproportionately impact tracking of these features, since missing one word may potentially distort neural computations of surprisal and lexical predictions for a large number of subsequent words. Note that this hypothesis does not discount the critical importance of lower level processes, such as those related to envelope processing, for speech comprehension. Instead, it suggests that the perceptual consequences of peripheral impairment may be most pronounced at the level of responses to high-level features.

Our observation of trends suggesting an association between the amplitude of the surprisal TRF in the N400 latency range and the performance on the comprehension questions suggests that surprisal may indeed reflect the extent of SIN perception difficulties. However, due to the exploratory nature of these analyses, and because similar trends were not observed for SSQ_m_, it remains unclear if this neuro-behavioral association is reliable. A follow-up study explicitly manipulating comprehension difficulty of speech materials while maintaining lower-level intelligibility (e.g., by presenting in- vs. out-of-order speech segments; see Broderick et al., 2020) may help to further explore this link.

It is notable that the correlations between SSQ_m_ or task performance and feature-specific model contributions were overall relatively weak in this study. Although this implies that none of the features utilized in our study can on their own predict the degree of SIN perception difficulties, it is possible that such deficits may be better characterized in terms of a multi-dimensional pattern of feature-specific neural responses. In other words, it may be the case that in order to predict the extent of SIN perception difficulties, a combination of neural measures across multiple lower- and higher-level speech features needs to be taken into account. Along these lines, Lesenfants et al. (2019) showed that speech reception thresholds can be predicted from EEG responses to speech more accurately using a model that contains both spectrogram and phonetic features, compared to models containing only one of the features. Furthermore, because SIN perception difficulties can have different underlying etiologies, with different relative contributions from peripheral damage and cognitive factors, it may be the case that distinct patterns of feature-specific responses characterize different underlying causes of SIN deficits.

### Limitations

Although our work provides evidence of age-related differences in cortical tracking of word-level features, a notable limitation of our method is that it does not establish the source of this difference. Specifically, it is unclear from our data if the distinct patterns of feature-tracking were a result of higher-order linguistic mechanisms receiving inputs with differing fidelities from lower-level processes, or they reflected age-related changes in the higher-order mechanisms themselves, or some combination of the two. Furthermore, differential engagement in cognitive resources (e.g., due to differential effort) may also have contributed to the observed differences, even in the absence of actual changes in the underlying mechanisms. Thus, an important goal for future work is to characterize speech representations more thoroughly at multiple levels of the processing hierarchy in order to elucidate the mechanisms implicated in the differences in speech processing. Furthermore, the measurement of speech representations at multiple stages of the language processing hierarchy may be critical for explaining individual differences in speech perception performance, and subjective measures such as the SSQ_m_.

Although the goal of the present study was to look for evidence of age-related changes in speech processing, it is notable that the distribution of ages in our older group did not extend into particularly old ages (e.g., only 5/21 of OA participants were older than 60 years). This may have contributed to the relatively small difference between groups in our data, and our inability to detect robust differences in the TRFs. Additionally, it is unclear how results from our OA sample would generalize to even older populations. While inclusion of older (65+ y.o.) participants is complicated by hearing loss that may be difficult to compensate for via amplification (e.g., due to hardware limitations and safety concerns), it is important for future work to better represent these participants in the OA sample. Additionally, the present study treated age as a categorical variable due to concerns that individual differences unrelated to age would prevent us from detecting age-related changes in analyses that treat age as a continuous variable. However, in order to gain more quantitative insight into *how* age is related to neural speech processing, it will be important to conduct larger-scale studies suited for treating age as a continuous variable.

The use of artificial neural networks (ANNs) to extract abstract features related to lexical and semantic content of speech has become increasingly common in studies of language processing (Huth et al., 2016; Broderick et al., 2018; Weissbart et al., 2019; Donhauser and Baillet, 2020). While powerful in characterizing brain responses to speech, an important limitation in the use of these features is that it can be difficult to interpret what aspects of language they actually capture. Specifically, ANNs are usually trained on a task such as text prediction on the basis of preceding context, and as such, ANNs may utilize any number of statistical regularities in the training corpus in order to optimize their performance. Thus, depending on the ANN architecture, aspects of language including the syntactic structure, lexical frequency, semantic relationships, and others may all contribute to the performance of ANNs. Without knowing the language aspects learned by ANNs, it is difficult, and may be even impossible, to parse out the relative contributions of the different variables. Consequently, when cortical responses are found to track these features, as is the case in the present study, it may remain unclear what linguistic processes underlie this tracking. Thus, improving the interpretability of neural analyses that utilize complex natural language models remains an important challenge for future work.

## Conclusion

The present study extends upon the existing body of work demonstrating the plausibility of measuring cortical tracking of high-level features related to speech meaning and predictability. The results show evidence of age-related amplification in tracking of these features in competing speech streams, albeit it remains unclear whether these differences stem from changes in speech processing mechanisms, differences in listening effort, or some other cognitive factors. Moreover, our exploratory analyses showed trends of correlations between these measures and behavioral measures including comprehension performance and subjective SIN perception difficulty scores, indicating their potential behavioral relevance. Taken together, our work demonstrates the utility of modeling cortical responses to multi-talker speech using complex, word-level features and the potential for their use to study changes in speech processing due to aging and hearing loss.

## Data Availability Statement

Data is not available publicly, as data sharing was not a part of the informed consent. Requests to access the dataset should be directed to JM, mesik002@umn.edu or MW, wojtc001@umn.edu.

## Ethics Statement

The studies involving human participants were reviewed and approved by Institutional Review Board of the University of Minnesota. The participants provided their written informed consent to participate in this study.

## Author Contributions

JM and MW designed the experiment, analyzed the data, and wrote the manuscript. JM and LR implemented the experimental procedures and collected the data. All the authors commented on the manuscript and approved the submitted version.

## Conflict of Interest

The authors declare that the research was conducted in the absence of any commercial or financial relationships that could be construed as a potential conflict of interest.
