# Supplementary material for: Effects of Age on Cortical Tracking of Word-Level Features of Continuous Competing Speech
Source: Front Neurosci. 2021 Apr 1;15:635126. doi: 10.3389/fnins.2021.635126 (PMC8047075; doi:10.3389/fnins.2021.635126)
Supplement: Supplementary file 1 [file Data_Sheet_1.PDF]

Supplementary Material

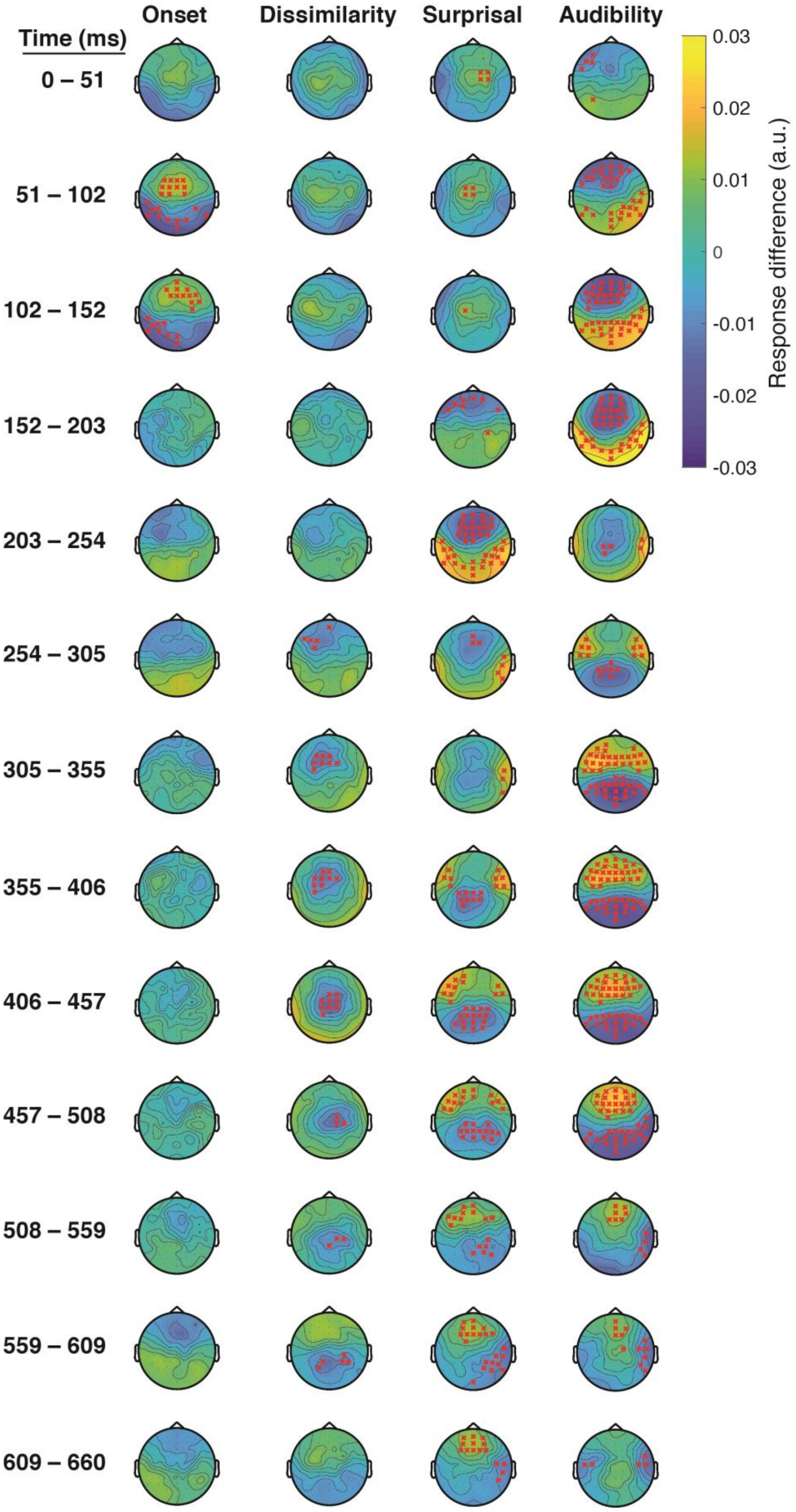

Onset
Dissimilarity
Surprisal
Audibility
Time (ms)
0 – 51
51 – 102
102 – 152
152 – 203
203 – 254
254 – 305
305 – 355
355 – 406
406 – 457
457 – 508
508 – 559
559 – 609
609 – 660
0.03
0.02
0.01
0
-0.01
-0.02
-0.03
Response difference (a.u.)

Supplementary Figure 1. Attentional modulation of feature-specific responses: Cluster-based permutation approach. Each row of scalp topographies depicts a time-course, in ~50 ms steps (13 samples/step = 50.8 ms), of the Attended - Ignored TRF differential for each of the four features. The red symbols denote electrodes that were included in spatio-temporal clusters that supported a statistically significant difference between the attended and ignored TRFs (α = 0.006, two-tailed comparison).

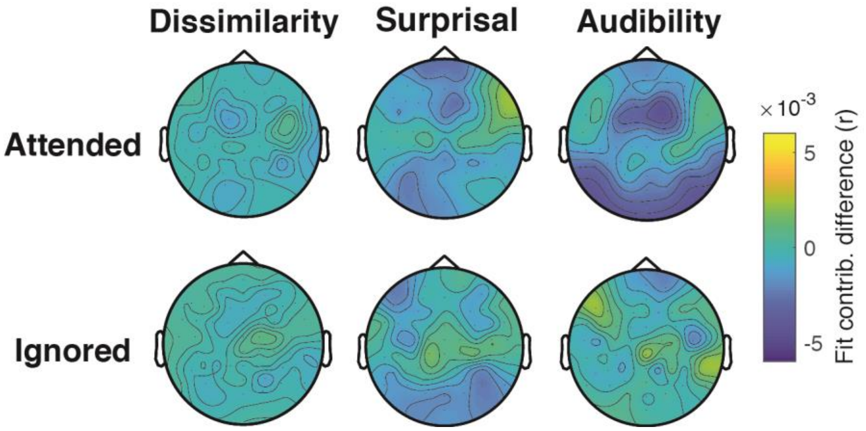

Supplementary Figure 2. Age group differences in feature-specific goodness-of-fit contributions: Scalp topographies depict the YA - OA differential between model goodness-of-fit contributions for each feature (columns of topographies), when speech was either attended or ignored (rows of topographies). Note that statistical analyses have not been computed on these topographies, so the differences serve descriptive purposes only.

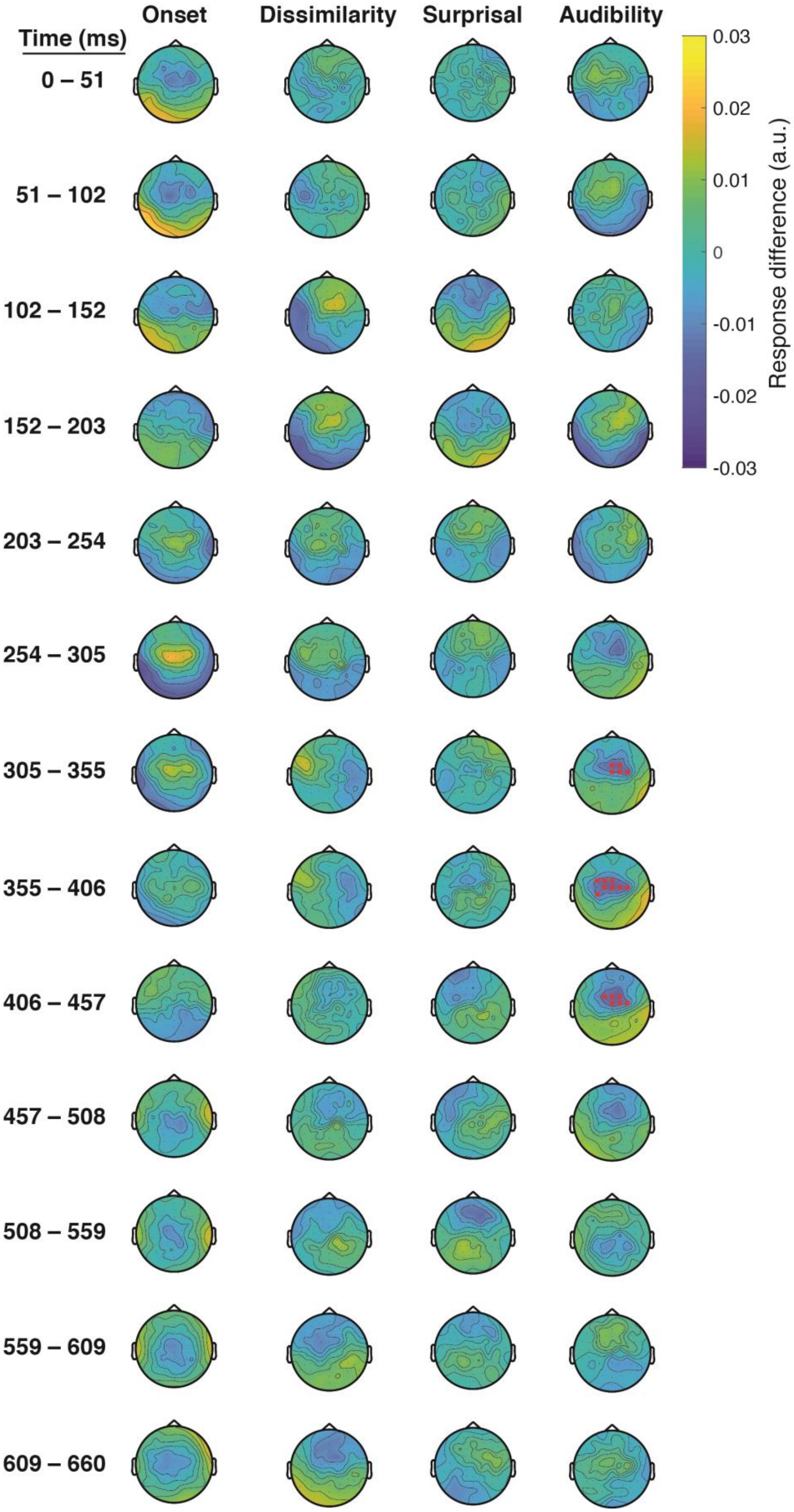

Onset
Dissimilarity
Surprisal
Audibility
Time (ms)
0 – 51
51 – 102
102 – 152
152 – 203
203 – 254
254 – 305
305 – 355
355 – 406
406 – 457
457 – 508
508 – 559
559 – 609
609 – 660
0.03
0.02
0.01
0
-0.01
-0.02
-0.03
Response difference (a.u.)

Supplementary Figure 3. Age group differences in feature-specific responses to attended speech: Cluster-based permutation approach. Each row of scalp topographies depicts a time-course of the YA - OA TRF differential for each of the four features. The red symbols denote electrodes that were included in spatio-temporal clusters that supported a statistically significant difference between TRFs from YA and OA ($\alpha = 0.006$, two-tailed comparison).

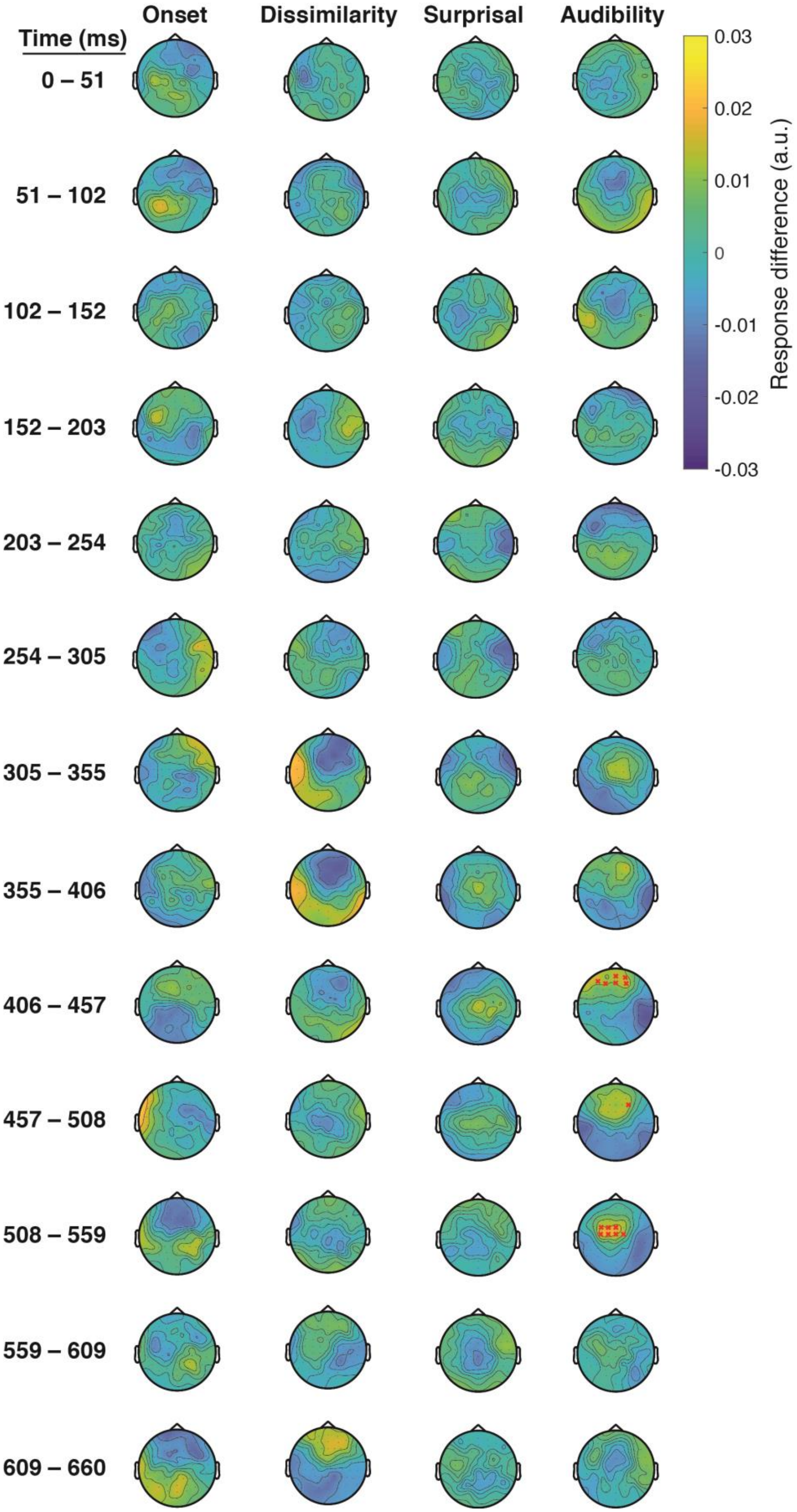

Onset
Dissimilarity
Surprisal
Audibility
Time (ms)
0 – 51
51 – 102
102 – 152
152 – 203
203 – 254
254 – 305
305 – 355
355 – 406
406 – 457
457 – 508
508 – 559
559 – 609
609 – 660
0.03
0.02
0.01
0
-0.01
-0.02
-0.03
Response difference (a.u.)

Supplementary Figure 4. Age group differences in feature-specific responses to ignored speech: Cluster-based permutation approach. Each row of scalp topographies depicts a time-course of the YA - OA TRF differential for each of the four features. The red symbols denote electrodes that were included in spatio-temporal clusters that supported a statistically significant difference between TRFs from YA and OA ($\alpha = 0.006$, two-tailed comparison).
